# Controversial Ebola vaccine trials in Ghana: a thematic analysis of critiques and rebuttals in digital news

**DOI:** 10.1186/s12889-017-4618-8

**Published:** 2017-08-07

**Authors:** Per Egil Kummervold, William S. Schulz, Elizabeth Smout, Luis Fernandez-Luque, Heidi J. Larson

**Affiliations:** 1 Norut Northern Research Institute, P.O. Box 6434, Tromso Science Park, N-9294 Tromso, Norway; 2 London School of Hygiene & Tropical Medicine, London, UK; 3 Qatar Computing Research Institute, Hamad Bin Khalifa University, Doha, Qatar

## Abstract

**Background:**

Communication is of paramount importance in responding to health crises. We studied the media messages put forth by different stakeholders in two Ebola vaccine trials that became controversial in Ghana. These interactions between health authorities, political actors, and public citizens can offer key lessons for future research. Through an analysis of online media, we analyse stakeholder concerns and incentives, and the phases of the dispute, to understand how the dispute evolved to the point of the trials being suspended, and analyse what steps might have been taken to avert this outcome.

**Methods:**

A web-based system was developed to download and analyse news reports relevant to Ebola vaccine trials. This included monitoring major online newspapers in each country with planned clinical trials, including Ghana. All news articles were downloaded, selecting out those containing variants of the words “Ebola,” and “vaccine,” which were analysed thematically by a team of three coders. Two types of themes were defined: critiques of the trials and rebuttals in favour of the trials. After reconciling differences between coders’ results, the data were visualised and reviewed to describe and interpret the debate.

**Results:**

A total of 27,460 articles, published between 1 May and 30 July 2015, were collected from nine different newspapers in Ghana, of which 139 articles contained the keywords and met the inclusion criteria. The final codebook included 27 themes, comprising 16 critiques and 11 rebuttals. After coding and reconciliation, the main critiques (and their associated rebuttals) were selected for in-depth analysis, including statements about the trials being secret (mentioned in 21% of articles), claims that the vaccine trials would cause an Ebola outbreak in Ghana (33%), and the alleged impropriety of the incentives offered to participants (35%).

**Discussion:**

Perceptions that the trials were “secret” arose from a combination of premature news reporting and the fact that the trials were prohibited from conducting any publicity before being approved at the time that the story came out, which created an impression of secrecy. Fears about Ebola being spread in Ghana appeared in two forms, the first alleging that scientists would intentionally infect Ghanaians with Ebola in order to test the vaccine, and the second suggesting that the vaccine might give trial participants Ebola as a side-effect – over the course of the debate, the latter became the more prominent of the two variants. The incentives were sometimes criticised for being coercively large, but were much more often criticised for being too small, which may have been related to a misperception that the incentives were meant as compensation for the trials’ risks, which were themselves exaggerated.

**Conclusion:**

The rumours captured through this research indicate the variety of strong emotions drawn out by the trials, highlighting the importance of understanding the emotional and social context of such research. The uncertainty, fear, and distrust associated with the trials draw from the contemporary context of the Ebola outbreak, as well as longstanding historical issues in Ghana. By analysing the debate from its inception, we can see how the controversy unfolded, and identify points of concern that can inform health communication, suggesting that this tool may be valuable in future epidemics and crises.

**Electronic supplementary material:**

The online version of this article (doi:10.1186/s12889-017-4618-8) contains supplementary material, which is available to authorized users.

## Background

In 2014 the Ebola outbreak in West Africa became a global health emergency, spurring an international effort to trial candidate Ebola vaccines and other potential prevention and control measures. The European Union Innovative Medicines Initiative (IMI) funded several projects to help combat the epidemic, one of them EBODAC (EBOla Vaccine Deployment, Acceptance and Compliance).

Within its broader communication and community engagement mandate, EBODAC addressed the risk of misinformation and controversies undermining the trial of a prime-boost Ebola vaccine regimen, developed by Janssen Pharmaceutical Companies of Johnson & Johnson (Janssen). Rumours were already hindering the Ebola response in the affected countries [1], [2] and the early detection and analysis of emerging rumours around the vaccine trials was considered a priority for EBODAC [3].

In February 2015, the EBODAC consortium launched a system for real-time monitoring and analysis of online newspapers in Sierra Leone, and in April 2015 the media monitoring system was extended to include Ghana, Uganda and Kenya, where Phase I trials for Ebola vaccines were also being planned.

In late May 2015, a controversy was reported in Ghanaian newspapers and became inflamed into a public debate, finally resulting in the suspension of both the planned Phase 1 trial for the Janssen Ebola vaccine at the University of Health and Allied Sciences (UHAS) in Hohoe, Volta region, and a planned Phase 2 trial for the GlaxoSmithKline (GSK) Ebola vaccine in Hohoe and in Kintampo. This controversy was exacerbated by the fact that, when these critical articles first became public, both the Minister of Health and the trial lead investigator were travelling outside Ghana – a fact which should be borne in mind while reviewing this analysis.

At the time the controversy broke out, the Ghana Food and Drugs Authority (FDA) was considering both vaccine trials for approval. Five months after the suspension, both vaccine trials were finally approved by Parliament [4]. To date, however, neither trial has proceeded since receiving this approval.

This article examines the messages and perspectives put forth by different stakeholders in the controversial vaccine trials in Ghana through the use of a media monitoring and analysis system. The study analyses the stakeholder concerns and incentives, and the phases and dynamics of the dispute. It provides a unique methodological approach to studying complex interactions between public health authorities, political actors, and public citizens, offering lessons for future research.

### Historical research controversies and their implications

History offers several examples of the importance of addressing stakeholder concerns in public health initiatives, since failure to do so can undermine health research and programmes, even when these concerns are rumour-driven [5–8].

In Nigeria in 2003, for example, Northern states boycotted polio vaccination campaigns, because of a complex set of factors including the memory of a controversial trial of the antibiotic Trovan in 1996, in which 11 children died [9]. Although the deaths were deemed to be unrelated to the antibiotic, the incident led to a multitude of lawsuits both in Nigeria and the USA, one of which ended in an out-of-court settlement of 75 million USD related to ethical misconduct, paid to the state of Kano in 2009 [10].

The Trovan trial left a lasting distrust in Kano. In 2003 a leader of the polio boycott justified his opposition to the vaccination campaign by referring to, “…the Pfizer scandal, when our people were used as guinea pigs with the approval of the federal ministry of health, and the approval of all the relevant UN agencies” [11]. A Kano farmer interviewed in 2005 put the issue more bluntly:

*“We cannot trust the white man or our federal government because many years ago they were in partnership… Our government does not have our interests at heart, that is why these people can come in any time they want and do whatever they want”* [12].

This perception that the government responds more to international interests than to its own people was reinforced by the fact that polio vaccines were being provided free by the Global Polio Eradication Initiative (GPEI), while services for other pressing health needs were lacking [13, 14]. The polio boycott, which allowed polio cases to quadruple between 2002 and 2006, re-seeded the disease in multiple countries that had already eliminated it, and cost the GPEI 500 million USD [15]. This case illustrates how important it is to recognize and address stakeholder concerns early, as they can influence public trust, with serious public health consequences in the long term.

## Methods

To monitor news in each country with planned vaccine trials – Sierra Leone, Ghana, Uganda, and Kenya – the project team selected online newspapers in each country. The selection was based on a qualitative assessment of expected impact and maximum variation to ensure detection of any news regarding the planned trials. Services like Google News Alert^1^ were considered for monitoring new articles meeting specific criteria, but in several of the target countries the major search engines indexed only a few of these news outlets, and these services were therefore not appropriate. Instead a PHP-based^2^ system was set up to monitor the front page of the outlets for new articles every hour, downloaded any new articles and scanned the articles for variants of the keywords “Ebola” and “Vaccination”. The system also forwarded all articles containing relevant keywords to the Ebola vaccine program communication crisis team.

Using a thematic analysis [16] approach, three independent coders (LL, WS, PEK) reviewed a subset of articles and were assigned to identify between 5 and 15 themes. Findings from a previous study [17] investigating online vaccine sentiments were used as a starting point for the codebook, which was further adapted to the Ghana context. The team met to compare their proposed themes and 24 were initially agreed upon, but after a change in coders (ES, WS, PEK) an additional three themes were added, for a total of 27 themes [see: Appendix 1].

Theme inclusion criteria were: a) they should be repeated in at least two different news outlets; and b) they should be able to be reduced to declarative statements about the trials or their proponents/opponents.

Two overarching types of themes were defined: (1) “critiques” which directly or indirectly argue that the trials should not take place, and (2) “rebuttals” refuting the critiques, sometimes introducing new arguments in favour of the trials.

Based on the codebook, a web-based coding interface was developed allowing the coders to mark example text and attach relevant coding tags to the text [see: Appendix 2]. When all coders had completed the first round of scoring, the coders unanimously agreed on 87% of the coding decisions. This gave an inter-rater reliability (IRR) score of 0.62, calculated using Fleiss’ kappa, which can be considered a high level of agreement [18]. The main reason for calculating IRR here is to improve reliability. Inter-rater disagreement can arise from actual disagreement, but when dealing with large amounts of data, it is often based on misreading or overlooking themes that are present.

In the second round, where one coder disagreed with the other two, s/he was presented with the other raters’ coding, accompanied by a relevant text extract from the article, and asked whether s/he agreed. If s/he still disagreed it was sent back to the others. This was repeated, and virtually all discrepancies were reconciled. After two rounds of the reconciliation process there were only five disagreements left, giving an IRR which was very close to 1.0. In these remaining five cases the coders agreed that the coding decisions were fundamentally subjective [see: Appendix 3 for more details on the coding procedure].

## Results

**Fig. 1:**
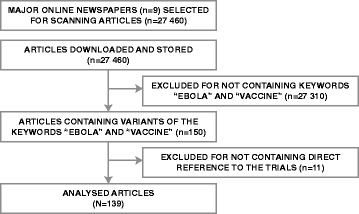
Study design flow chart. Flow chart shows data sources and exclusion by keyword search and thematic analysis

A total of 27,460 articles from nine different newspapers were collected on-line from Ghana in the time period from May 1st to July 30th 2015. Of these, 150 articles in six different newspapers contained the keywords. Eleven of the 150 articles were excluded because they referred only indirectly to the trials, or not at all. The final dataset included 139 articles (Fig. 1) [see: Appendix 3 for a list of analysed articles].

**Fig. 2:**
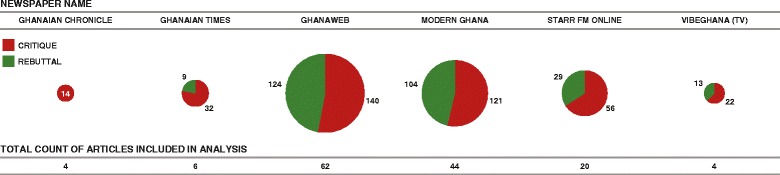
Overall count of articles included from each publisher, with theme proportions. Diagrams show the proportion of negative and positive themes (critiques and rebuttals) in articles from each publisher, with size scaled to represent the number of articles included from each news outlet (shown also in tabular form beneath)

The 139 reports analysed were collected from six news outlets (Fig. 2) in which we identified 385 instances of critiques and 279 instances of rebuttals.

Because the codebook specified more negative themes than positive themes, no conclusions should be drawn about overall ratio of positive versus negative sentiment. Instead, we gain insights by tracking groups of related themes over time, how they evolved, identifying which critiques were more prevalent, and which rebuttals were issued in response. There were three key areas of critique: (1) the allegation that the trials were secret; (2) the belief that the trials would lead, in one way or another, to Ebola cases in Ghana; and (3) the argument that the trials were providing inappropriate incentive packages for participants.

**Fig. 3:**
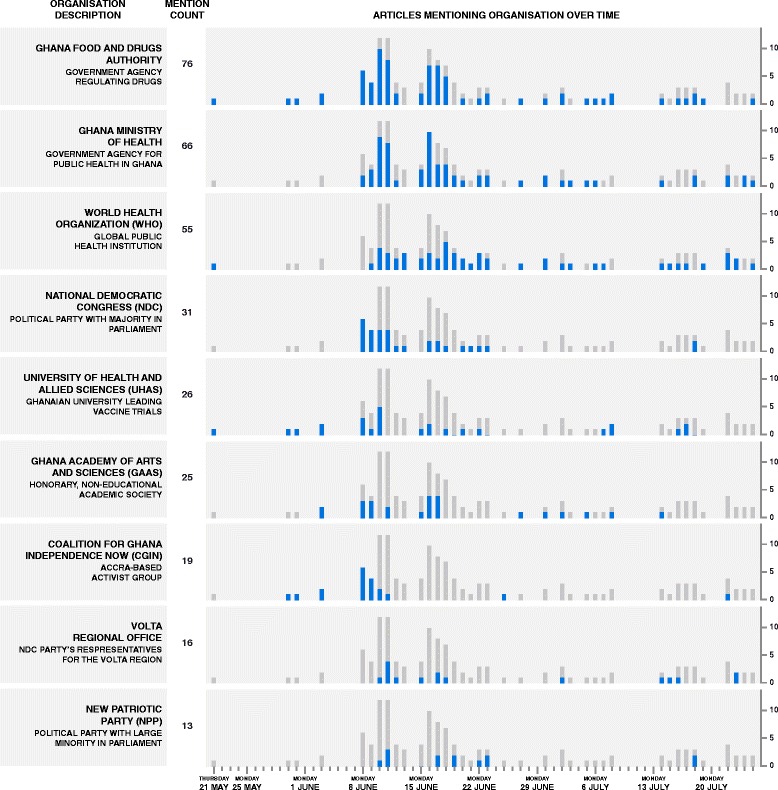
Daily count of stakeholder mentions. Number of articles mentioning institutional stakeholders daily (*blue bars*), compared to total relevant articles daily (*grey bars*). Quantified by keyword search, conducted after completion of thematic analysis

Figure 3 shows the major organisations, listed in order from most-mentioned to least-mentioned, referenced in the media reports about the trials. Blue bars indicate the number of articles mentioning a given organisation shown against grey background bars representing the total number of included articles published on a given day. The Ghana FDA was named in the very first articles in mid-May and remained a key actor throughout the debate, whereas the Ministry of Health and World Health Organisation (WHO) became more involved in mid-June. Early and ardent critics of the trials, such as the Coalition for Ghana’s Independence Now (CGIN) and the National Democratic Congress (NDC), were most involved in early June, whereafter their salience faded. Meanwhile the Ghana Academy of Arts and Sciences (GAAS) maintained a low but persistent media presence through to early July.

**Fig. 4:**
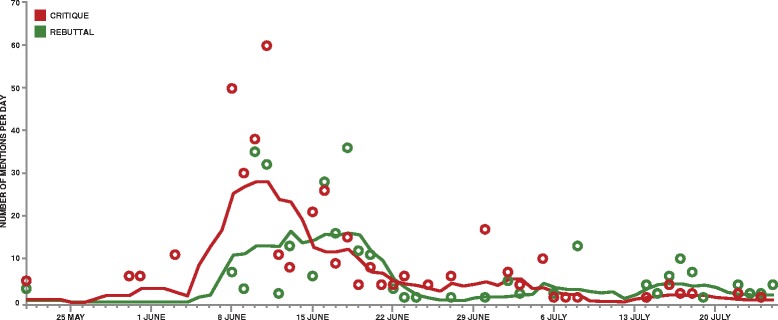
Daily combined count of critiques and rebuttals, exact and moving average. Legend: *Circles* indicate exact count of critiques (*red*) and rebuttals (*green*) appearing daily. *Line plots* show a 7-day moving average, calculated to show overall trend in critiques and rebuttals per day

Figure 4 shows the prevalence of positive and negative themes over time. The first article in our dataset was published 21 May 2015 by Starr FM Online, the website for the Ghanaian radio station Starr FM. This article broke the story, presented as an “undercover” investigation into a secret trial, which remained a common theme throughout the controversy (see section "Secret trial and insufficient public information"). It also drew attention to the incentives offered for participation in the trial, which likewise remained relevant for many weeks (see section "Incentives").

The story went seemingly unnoticed for more than a week, until a press release was issued by the CGIN.^3^ The statement (see Additional file 1), first published by Starr FM Online (30.05), claimed that the trial researchers would infect healthy Ghanaians with Ebola in order to test the vaccine (see section "The fear that vaccine trials will bring Ebola").

The story received increasing coverage over the following week, until the Volta regional office of the NDC political party issued a statement opposing the trials, which was then raised in Parliament. At this point, critiques spiked dramatically, and rebuttals followed.

**Fig. 5:**
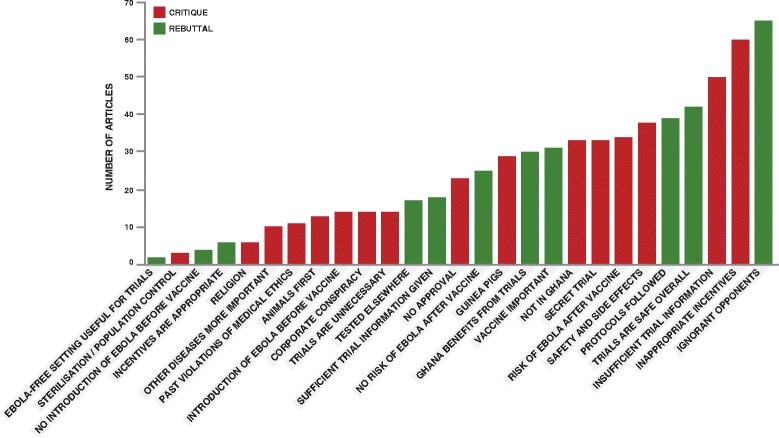
Overall count of theme mentions. Number of articles mentioning each theme, colour-coded to distinguish critiques (*red*) and rebuttals (*green*)

As shown in Fig. 5, a variety of themes were prominent in the ensuing weeks, including arguments that the trials were “unnecessary” because Ghana was unaffected by the Ebola outbreak, that other diseases were more important (Starr FM Online, 08.06a), and that the trials should be conducted in a country other than Ghana.

Rebuttals tended to be broader statements, making the case that the risk to participants was small and that there had been no deviation from established protocols (GhanaWeb, 08.06a), emphasising the global importance of developing an Ebola vaccine (GhanaWeb, 08.06a), and also that Ghana had an interest in the publicity and economic benefits of hosting such research (Modern Ghana, 16.06b). They also pointed out that the vaccines had already undergone some safety checks as they had been tested in other countries (GhanaWeb, 08.07).

The issue of “ignorance” emerged in a prolonged back-and-forth between Professor Alex Dodoo and the Ghanaian Parliament, during which Dodoo was summoned to explain his alleged criticism of Members of Parliament (MPs) as “ignorant” regarding the procedures of clinical trials.^4^ While this prompted considerable reaction, most of the related articles were as much about Parliament itself as they were about Professor Dodoo’s comments.

**Fig. 6:**
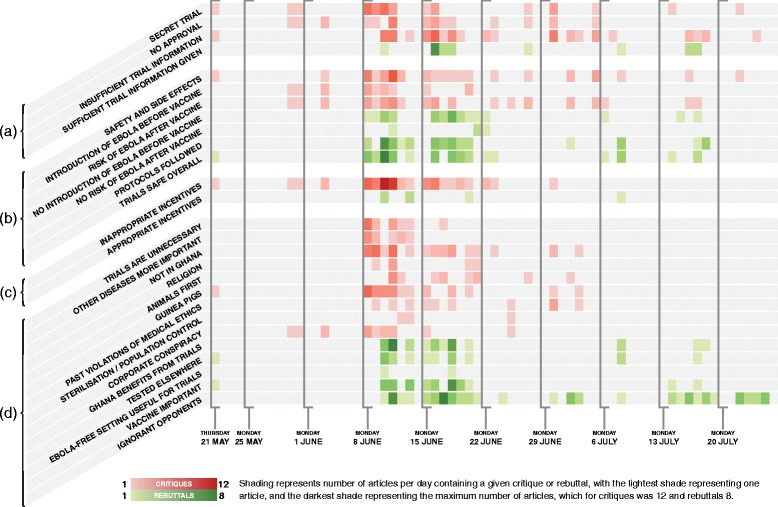
Daily count of individual themes, shaded by number of articles per day mentioning a given theme. Themes were conceived as “statements about the trials,” and divided into two categories: critiques of the trials (*red*) and rebuttals on behalf of the trials (*green*). **a** Themes discussed in section "Secret trial and insufficient public information": Descriptions of the trials as “secret” or “clandestine” were most prevalent in the first half of the controversy, but following Parliamentary discussions, this theme was superseded by the more restrained judgements that the trials had provided “insufficient information” to politicians and had failed to “sensitise” the general public. Trial representatives began responding in mid-June that information had been provided and public sensitisation would be done. **b** Themes discussed in section "The fear that vaccine trials will bring Ebola": Early concerns about the trials’ safety included two related but distinct fears – first, that trial participants might contract Ebola from the vaccine, and second, that because Ghana had no Ebola cases, the researchers must have intended to introduce Ebola into the population for the purpose of testing the vaccines. Rebuttals included repeated explanations of why the trial vaccines could not infect participants, as well as several assurances that Ebola was not being introduced to the country, however most rebuttals focused more on the idea that the trials were “following all protocols” and safe in a general sense. **c** Themes discussed in section "Incentives": Incentives – 200 Ghanaian Cedis (GH₵) and a mobile phone – were a target for extensive criticism, both from those who felt these were valuable enough to be coercive, and also from those who perceived the trials to be very risky and consequently viewed the incentives as insultingly small. Rebuttals included the clarification that phones were intended to facilitate communication between researchers and participants, and the money was to compensate participants for their time. **d** Uncategorised themes

A chronological overview of the major events can be found in Appendix 1. To give a visual summary of the coded data, a timeline of theme density is shown in Fig. 6.

## Discussion

### Secret trial and insufficient public information

Secrecy was a strong and recurrent theme (Fig. 6a), and 29 (21%) of 139 articles contained statements about the trials being a secret. The initial Starr FM Online article of 21 May included the passage, “*Investigations by Starr FM under-cover team have made public a clandestine attempt by authorities to use midwifery students at Hohoe in the Volta region for human experiment on the Ebola vaccine in a country with no Ebola case.”* The CGIN press release brought greater attention to this article, and this specific passage is repeated in a total of 19 articles up until 8 June.

Descriptions of the trials as “secret” or “clandestine” were most prevalent in the first half of the controversy, but following Parliamentary discussions, this theme was superseded by the more restrained judgements that the trials had provided “insufficient information” to politicians and had failed to “sensitise” the general public. Trial representatives began responding in mid-June that information had been provided and sensitisation would be done.

This softer criticism, that the trials had provided “insufficient information,” appeared in 39 (28%) of the articles. In an article titled, “House orders ‘secret’ Ebola vaccination stopped,” MPs’ objections focused on a lack of consultation with politicians and the general public:

*“(…) the Deputy Minister of Education, Samuel Okudzeto Ablakwa, said it was unacceptable for the ministry to carry out such a sensitive exercise without proper communication and sensitisation. He said the exercise (…) did not have political approval from the government, and appealed to his colleagues not to put the blame on the government.”* (Ghanaian Times, 11.06).

Speaking before Parliament, Health Minister Alex Segbefia struck a contrite tone, admitting the need for better public engagement but asserting that approval had been sought from all the appropriate institutions:

*“He said standard protocol had been followed prior to the approval for the vaccination to take place, but he conceded that despite the rigorous nature of the approval process, the stakeholder consultation that needed to have been done was not thorough enough.”* (GhanaWeb, 16.06a).

In an interview published the following day, however, Susan Adu Amankwah (a researcher unaffiliated with either trial) ridiculed parliamentarians’ expectation of being personally consulted:

*“The protocol of the trial had gone through the FDA … [in accordance with] the law that they [MPs] themselves passed – the Public Act 815 so the government knew about it. If the government is not represented by the FDA, if the state is not represented by the FDA, then I don’t know who the FDA is.”* (GhanaWeb, 17.06b).

Even as new public education efforts got underway, the secrecy claim continued to reappear, such as when the NPP party Ashanti Regional Chairman used it to direct blame at Ghana’s president,

*“… wondering how government, under the leadership of President John Dramani Mahama, could allow such an exercise to be conducted in his homeland, without the necessary education and approval.”* (Ghanaian Chronicle, 22.06b).

In an article titled “When Scientists Become Too Secretive,” author Cameron Duodo was clearly unconvinced by the legalistic argument that the trials had received proper approval:

*“… scientists of the Ghana Food and Drugs Authority (FDA) blithely authorised Ghanaian scientists, working for a foreign pharmaceutical company to carry out trials of an Ebola vaccine, without so much as a word to the Ghanaian public, to prepare their minds for the trials.”* (Ghanaian Times, 30.06).

This narrative of an immoral experiment lent itself to historical comparisons, including the Tuskegee Syphilis Experiment (Modern Ghana 08.06), its Guatemalan counterpart (Ghanaian Chronicle, 25.06), and medical crimes perpetrated under the racist regimes of Nazi Germany and Apartheid South Africa (Ghanaian Times, 30.06). While such references were relatively sparse, Ghana’s colonial past is a recurring theme throughout the debate, particularly in the “incentives” issue.

Lastly, long after the peak of the debate, a key traditional leader in the Hohoe region professed his longstanding support for the trials, and his bemusement at the national controversy:

*“The Paramount Chief of Gbi Traditional Area, Togbe Gabusu, … wondered why the Ebola vaccine trial should generate so much controversy. “I have never doubted this because when they [researchers] came, they called all the traditional heads. They met us talked to us and we were satisfied before the hullabaloo came. We have a Research Centre in town here, it has been here for [many] years…and they have been working. So why this?”, the Paramount Chief wondered.”* (Modern Ghana, 17.07).

The idea that the trials were “secret” was initially propagated by the Starr FM Online article, in which local students described being approached about participating in an upcoming trial, and in which interviewees affiliated with the UHAS research centre “pleaded anonymity,” and insisted they “could not disclose” information. It seems likely that the interviewees were attempting to adhere to perfectly ordinary non-disclosure agreements, considering that the trials were still under review. This may have added an unintentionally conspiratorial air to their comments.

The Starr FM Online article also instigated the idea of secrecy by the mere fact that it was the first place where the trials were widely publicised. In the words of commentator Cameron Duodu,

*“if you … try, even if metaphorically, to “smuggle” the project into the country, you will ensure that the first that is heard of it is through the news broadcast by a local radio station, then you are asking for trouble”* (Ghanaian Times, 30.06).

The mere fact that the public was not aware of the trials lent credibility to the claims in the Starr FM Online article. Then again, the trials were forbidden to conduct any public education prior to receiving ethics approval, so its absence was not so much a mistake, but rather a vulnerability inherent in the established protocol for conducting research, which may need to be revised.

### The fear that vaccine trials will bring Ebola

One of the most common critiques was that the vaccine trials would lead to Ebola cases in Ghana. Out of 139 articles in our dataset, 47 (33%) claimed that the vaccine would cause an Ebola outbreak in Ghana, while 27 articles (19%) said this would not happen. The critique came in two distinct forms: first, a suspicion that trial researchers would intentionally expose people to Ebola virus in order to test the vaccine, and second, a fear that the vaccine itself would cause participants to contract Ebola. Rebuttals included direct responses to both of these critiques, and overarching statements that the trials were safe and protocols had been followed. Figure 6b shows when each of the critiques appears, and when each is countered.

The claim is first mentioned in Starr FM Online (30.05), and is a direct quote from a press release issued by CGIN:

“*Ebola outbreak, which is 100% sure to happen in Ghana should this human trials be allowed to go on, will be the greatest national security threat our country will ever face. The manufacturers of the so-called Ebola vaccine will look on till a larger number of Ghanaians are killed by the disease here in Ghana before the vaccine will be released and this would happen to make government buy the vaccine at any price…there is and will be no way by which Ghana can go through this Ebola virus human experiment without Ebola being spread country wide*.” (Starr FM Online, 30.05).

Thus the original claim was that Ebola had to be introduced to be able to test the vaccine. After a fierce debate, in which the CGIN statement was referenced in the majority of the monitored newspapers, the first statements defending the trials found their way into the media more than a week later:

“*The Food and Drugs Authority (FDA) has refuted claims that the impending Ebola vaccine trial in Ghana will harm persons who will be used as subjects for the exercise.* ”(Starr FM Online, 08.06).

“*According to FDA documents, the vaccines to be tested in Ghana are made using a common cold virus called an adenovirus that “does not make people sick”. The vaccines contain extracts that do not cause the disease from the Ebola virus*.” (GhanaWeb, 09.06).

After the trials were suspended by the Minister of Health on 9 June (after being ordered to do so by Parliament) the debate continued and the GAAS issued a press statement on 12 June stating that it was “unsafe to undertake the trials in Ghana”. The statement included a list of ten technical questions, including:

*“What assurances do we have that the chimpanzee-derived live adenovirus vector used in the GSK vaccine construct, although non-replicating for now, will remain dormant and not itself cause a disease to compromise the health of the people of Ghana?”* (GhanaWeb, 15.06a).

Several writers demanded that “The World Health Organization Must Respond to the Queries of the Ghana Academy of Arts and Sciences,” (GhanaWeb, 27.06). On 26 June UHAS issued a press statement [19] rebutting the scientific questions point-by-point. Principal Investigator Professor Fred Binka also gave rebuttals in person at a sensitisation forum, and offered to undergo vaccination himself (Modern Ghana, 22.06). However, newspapers continued re-printing the GAAS critiques as late as 5 July, without acknowledging these rebuttals. Only one article reported on the UHAS response, and not until 8 July.

Moreover, the GAAS was portrayed as a mouthpiece of the people: “Ghanaians, through the Ghana Academy of Arts and Sciences (GAAS), have posed Ten Queries which WHO is required to take extremely seriously and reply to.” (GhanaWeb, 27.06), in contrast to portrayals of the Ghana FDA as “…a body grandiosely semi-labelling itself after an illustrious American name sake…” (GhanaWeb, 15.06).

So, it appears that the initial fear about scientists intentionally introducing Ebola was gradually replaced by the concern that the vaccine itself could cause disease, and this narrative became somewhat more scientifically sophisticated after the GAAS’ list of questions became a focal point. There were other passages, such as “*The Ho West MP added that MPs from the region have been inundated with phone calls from panic-stricken constituents who believe that this trial is aimed at spreading the dreaded Ebola disease in the Volta Region*. ”(GhanaWeb, 15.06b), that indicate a perception that people would be infected prior to vaccination. However, most quotes between 16-22 June are related to whether the adenovirus vector might mutate into a virulent form of the Ebola virus and infect trial participants.

### Incentives

The debate around incentives was challenging to respond to: most complained that the incentives for trial participation were insufficient, but others simultaneously claimed they were inappropriately large, and in general the allegations made no distinction between the GSK trial (which was to offer participants money and a mobile phone) and the Janssen trial (which was not). The incentives were perceived as compensation for the trial’s dangers (as opposed to compensation for the cost of travel and time lost), and these dangers were thought to be great, leading to the conclusion that the incentives were too little. There were far fewer concerns about the incentives being coercively large compared with the many allegations that they were insultingly paltry. The claim that these incentives violated international standards for participant compensation, in some places attributed to WHO (see for instance GhanaWeb, 10.06d), added to the sense of insult. Rebuttals were issued to the effect that the incentives were for practical purposes (money for transport and phones for follow-up communication), but it is evident that the density of these rebuttals was sparser than that of the critiques.

The “incentives” theme appeared early in the debate peaking in the month of June (see Fig. 6c). It is one of the most frequent themes, with 49 of 139 articles (35%) mentioning it, and received relatively few rebuttals.

Incentives – 200 Ghanaian Cedis (GH₵)^5^ and a mobile phone – were a target for extensive criticism, both from those who felt these were valuable enough to be coercive, and also from those who perceived the trials to be very risky and consequently viewed the incentives as insultingly small. Rebuttals included the clarification that phones were intended to facilitate communication between researchers and participants, and the money was to compensate participants for their time and transport.

As with secrecy, the topic of incentives is central in the initial Starr FM Online article:

*“Documents cited by* *Starrfmonline.com* *indicate that the students have been promised [GH₵] 200 each and mobile phones.” They will also receive other compensations (…) “I’m really scared and a lot of my colleagues are apprehensive too,” a student confided in* *Starrfmonline.com* *. “Currently, they are compiling our names for the trial but we don’t know whether the vaccine is safe or not; whether we’ll contract the disease or otherwise. Nobody is explaining anything to us.”* (Starr FM Online, 21.05).

The incentives are described neutrally, yet appear alongside fearful quotes. Similar references appeared in late May and early June, until the Volta office of the NDC party released a statement condemning the trials and portraying the incentives in a decidedly negative light:

*“We are appealing to all Voltarians to remain calm and should not risk their lives for [GH₵] 200 and mobile phone,” a statement signed by the NDC regional chairman Kwadwo Gyapong, secretary Simon Amegashie-Viglo and regional organiser Henry Kojo Ametefee said.* (Starr FM Online, 08.06a).

In an opinion piece published the following day, writer Michael Bokor issued a strong condemnation:

*“Clearly, using Ghanaians as guinea pigs for this Ebola vaccine experiment is insulting and misguided. It is unethical, immoral, and despicable, especially if we consider what is being used as an inducement for participants.” (Modern Ghana, 09.06b).*

Not all articles were so critical, and at least one correctly reported that the phone was provided *“to facilitate communication and monitoring”* (Modern Ghana, 10.06c).

When the issue was brought before Parliament, however, the Majority Chief Whip proposed “*to invite the foreign pharmaceutical companies to do some explanations, because entering into a community and using mobile phones and [GHC] 200 to entice people for such a dangerous research is simply unacceptable*.” (Ghanaian Times, 11.06). Harsh opinion articles followed, arguing both that “*volunteers in other countries will not take less than US$2,000 or £1,000 per a single trial, in Ghana volunteers are being offered £40 ([GHC] 200) plus a cheap or substandard mobile (cell) phone likely to be of Chinese-made” (Modern Ghana, 12.06b)*, and also that that the incentives were coercively high and that participants:“*… should not be influenced in their decision by psychological or financial pressures of any sort.” (Ghanaian Times, 16.06).*

These contradictory criticisms presented an obvious challenge for defending the incentives. Early rebuttals focused on the practicality of the incentives:

*“…the [GHC] 200.00 was supposed to cater for the transportation of the volunteers while the mobile phone was to help health personnel assess the health of the volunteers once the vaccine has been administered on them.” (Modern Ghana, 10.06b).*

WHO country representative for Ghana Dr. Magda Rabalo managed to address the issue of the amount provided in the incentive package during a forum in Accra:

*“…the ethics committee of trials said such moneys should not be too much or too little to manipulate people to volunteer… It also noted that WHO does not define how much participant should be paid and that compensations differ from country to country.” (Vibeghana (TV), 20.06).*

Finally, several weeks later, an interview with Dr. Ama Kyerewaa Edwin, member of the Ghana Health Service Ethics Committee, clarified the actual compensation being given:

*“... [participants] are not paid for volunteering but you will be compensated for your time and traveling in and out of the research Centre. … Persons who offer themselves for the Ebola vaccine trial in Ghana will be given a comprehensive international insurance cover against any unintended consequences,” (Starr FM Online, 08.07).*

#### Limitations

This analysis only looks at English-language online media in a lower-middle-income country. It does not include print-only publications, radio or television broadcasts, and is therefore only analysing a subset of the media. However, we find few, if any, references in the online material to print-only sources.

In using these data to study a public debate, we assume that the timing and quantity of news coverage around a specific theme reflects the salience of that theme in the public consciousness. However, topics reported also reflect other factors, like editorial bias and preference for sensational stories.

As noted in the Results section, we make no attempt to draw conclusions from the aggregate amount of positive and negative themes observed in the dataset. A weighted analysis might account for the different number of positive and negative themes defined in the codebook. However, this would mask the fact that the themes do not measure discrete units of “positivity” and “negativity” but rather points and counterpoints in a narrative, which don’t combine additively. This may be an interesting subject for future research, nonetheless.

In our study we assume all articles to have equal influence. If the dataset were larger, it would be reasonable to attribute a weight based on the number of readers or geographic reach of a publication to estimate their potential influence.

Our analysis is based on manually coding themes. All coding was done using online tools, annotating the live data directly. Compared to traditional qualitative analysis, it is effective. However, it still means all articles matching specific keywords need to be read manually. Advancements in machine learning approaches might make it possible in the future to develop tools that will automate part of the coding in order to better provide real time monitoring to help health communication teams.

## Conclusion

The rumours identified in this dataset highlight the vital importance of understanding the emotional context of public concerns: a story of secret vaccine trials in Ghana, or scientists intentionally infecting Ghanaians with Ebola, is only credible in the context of deep mistrust. Indeed, the trials debate is marked, from beginning to end, by the overwhelming fear engendered by the Ebola outbreak, as well as a pervasive anxiety to distance Ghana from the disease, even at the expense of research that could help protect against it. Since the trials themselves were partly sponsored by Western pharmaceutical companies, opposing them could be portrayed as a show of independence from the influence of former colonial powers. In this way, critics tapped into long-running historical themes, as well as the visceral fear of the Ebola epidemic.

By analysing the debate from its inception, we can track the evolution of issues from their initial appearance in alarming news articles, to examples of extreme claims becoming more sober, as seen in the transition from claims of a “secret trial” towards more moderate complaints that too little sensitisation and stakeholder consultation had been done. As early opponents like the CGIN dropped out of the news, attention focused on high-profile figures, and in this process, these public figures may have felt that they needed to justify their initial position to avoid embarrassment. This could explain the overall increase in the sophistication of arguments against the trials, while the initial emotions remained at the heart of the debate. As shown in this analysis, even marginal critics can mobilize a widespread and sophisticated opposition movement to derail important public health programmes, if they are taken up by high-profile opinion leaders. The situation in Ghana demonstrates the importance of early and constant engagement with both supporters and critics of trials, bringing them into conversations and ensuring that their fears, concerns and perspectives are heard and considered from the very beginning of the process. We recommend early engagement as a way to minimise the risk of individuals and communities feeling excluded, or their concerns dismissed, provoking perceptions of secrecy and potentially leading high-profile opinion leaders to take up their arguments on a wider scale.

It is noteworthy that although some of the initial critiques remained major themes throughout the debate, others, such as the conspiratorial allegation that “Ebola is a business,” did not last as long. Not unlike an infectious disease outbreak, beginning with an isolated index case and followed by a larger flare-up, some strains of these rumours proved more contagious than others. New arguments and angles also emerged as more people joined the debate. For example, the controversy clearly resonated with racial and colonial themes, as evidenced by the repeated references to historical instances of unethical experimentation on African bodies. This should serve as a stark reminder that past injustices can still undermine trust in the present. In addition, researchers should work to build trusting relationships within the communities where they are working by being consistently open and transparent as early as possible. This is particularly vital when considering sensitive issues or protocol requirements such as taking blood or the use of incentives that may be particularly sensitive given histories of extraction in the region.

Although regulations sometimes prevent trials from sharing public information about a specific trial prior to receiving ethical approval, this doesn’t necessarily preclude the possibility of a more general, government-led national discussion on the need for an Ebola vaccine, the strong value that such a vaccine would have for Ghana’s national security, and the long-term economic and diplomatic benefits of taking part in the international effort. Such a generalised national dialogue - avoiding the specifics of any one candidate vaccine trial - should be considered, especially when the disease in question is so emotionally fraught, and so relevant to the whole nation. We also recommend the involvement of community representatives in the development of clinical trial protocols, in order to ensure that trial designs are both scientifically valid and also acceptable to the communities within which they are occurring.

One of the other major challenges is making the collected information comprehensible and useable to decision makers. To achieve this, effective visualisation techniques are vital. Some of these techniques are demonstrated in this article, and many of these can be generated on-demand in real time. For stakeholders involved in a major health crisis, real-time insights can be vital, given the limited amount of time available for decision-making. It is our hope that these techniques can be incorporated into a reliable tool for assessing public information needs in future epidemics and similarly emotive national crises.

## Appendix 1

**Table 1:** Final coding guidelines

Theme	Description	Type
Secret trial	Anything that indicates that the trial is/was secret.	NEG
No approval	The trial lacks approval from a public authority.	NEG
Inappropriate incentives	The incentives for trial participants are mentioned as a problem.	NEG
Insufficient trial information	The trial ought to have declared itself to / done more outreach and sensitisation with participants/general public/local leaders/national leaders. Having gotten approval without informing goes into this category	NEG
Corporate conspiracy	The Ebola/vaccine/trial being done for business.	NEG
Introduction of Ebola before vaccine	Any claim that the trial will *give people Ebola first, to be able to test the vaccine*	NEG
Risk of Ebola after vaccine	People will get Ebola *from the vaccine*	NEG
Safety and side effects	Any side effects and safety concerns	NEG
Past violations of medical ethics	The trial is similar to historical examples of unethical medical practice/experiments	NEG
Sterilisation/population control	The trial is part of a conspiracy to sterilise people / control the population	NEG
Other diseases more important	Addressing other health issues (e.g. cholera) more important than conducting the trial.	NEG
Not in Ghana	Thinks it *should* be done, but in *another place*, for reasons including: NIMBY/county/town/region/ethnic group, doesn't make sense because Ghana is Ebola-free	NEG
Guinea Pig	Any description of trial participants as Guinea Pigs.	NEG
Animals first	The vaccine hasn’t been tested on animals yet.	NEG
Trials are unnecessary	The trial is unnecessary/needless (e.g. because Ghana is Ebola-free)	NEG
Religion	The trial should not be done because “we prayed and God spared us” from Ebola	NEG
No introduction of Ebola before vaccine	We don’t have to give people Ebola to test the vaccine	POS
No risk of Ebola after vaccine	People will not get Ebola from the vaccine	POS
Protocols followed	Responses that say ethical, legal requirements have been met (which presumes public trust in the FDA, in the regulations and their enforcement). Excellent example of the difference between what ethics means to a researcher vs. a layperson.	POS
Vaccine important	Helping develop the vaccine is an ethical imperative in itself	POS
Ghana benefits from the trials	Participating the trials is in Ghana’s self interest since it will develop scientific and business capacity/reputation, and/or having the vaccine will help Ghana prepare for another outbreak	POS
Tested elsewhere	Other countries have tested the vaccine/are also testing it, which should reassure/inspire Ghana to participate as well	POS
Sufficient trial information given	We are doing/have done/ will do all the necessary and appropriate informing and sensitizing and outreach	POS
Ignorant opponents	People opposing the vaccine are ignorant etc. Including general characterisation of people opposing trial	POS
Appropriate Incentives	Money and phone are for practical purposes of transport and communication, not an undue incentive	POS
Trials are safe overall	The trial is safe (this code applies to all safety/side effect reassurances except those focusing on the fear of getting Ebola, which were coded as Not Ebola first/after)	POS
Ebola-free setting useful for testing vaccines	The test must be done in Ghana *because* it is an Ebola-free country	POS

## Appendix 2

Procedure for coding and resolving disagreements

***The following procedure were followed for each article after initial coding:***

- A theme that all coders included was marked as “present”.
- A theme none of the coders included was marked as “not present”.
- A theme *Coder 1* marked but not *Coder 2* or *Coder 3* was marked as “Challenge for Coder 1”.
  - ○ [Repeated for *Coder 2* and *Coder 3*]
- A theme *Coder 2* and *Coder 3* marked but not *Coder 1* was marked as “Challenge for *Coder 1*”.
  - ○ [Repeated for *Coder 2* and *Coder 3*]

***After initial coding all themes in the article then one of the following statuses:***

- Theme is present
- Theme is not present
- Challenge for *Coder 1*, *Coder 2* or *Coder3*

***Coder 1 was then presented with the following choice:***

- Coder 2 and Coder 3 marked this as theme X. They based this on the following quotes. Do you agree or disagree?
- Only you coded this as theme X. You did base it on the following quote. Do you still agree?
- [Repeated for *Coder 2* and *Coder 3*]

As long as the article was marked as a challenge, it was hidden for the agreeing coders. In cases where the challenge was not solved by agreement, it was sent back to the other coders, and the process repeated. The majority coders would then see one of message like:

- Coder 1 insists this should (not) be coded as theme X. Do you agree?

## Appendix 3

**Table 2:** Analysed articles and major incidents

Date	Newspaper	Url	Archived url	Title
21.05	This is the real start of the media debate. First article appears, but is not quoted by any other outlet until 10 days later			
21.05	Starr FM Online	http://www.starrfmonline.com/1.3920389	https://web.archive.org/web/20150524021135/http://starrfmonline.com/1.3920389	Ebola vaccine trial hits Hohoe; GH¢200, phones for participants
29.05	CGIN posts press release. Press release included as Additional file 1.			
30.05	Starr FM Online	http://www.starrfmonline.com/1.4162568	https://web.archive.org/web/20150602053125/http://www.starrfmonline.com/1.4162568	Stop “criminal” Ebola vaccines trial in Ghana - Coalition
31.05	GhanaWeb	http://www.ghanaweb.com/GhanaHomePage/NewsArchive/artikel.php?ID=360308	https://web.archive.org/web/20151025012812/http://www.ghanaweb.com/GhanaHomePage/NewsArchive/artikel.php?ID=360308	Stop “criminal” Ebola vaccines trial in Ghana - Coalition
03.06	According to article in the BMJ (http://www.bmj.com/content/350/bmj.h2105/rr-7. Archived at: https://web.archive.org/web/20170728081845/http://www.bmj.com/content/350/bmj.h2105/rr-7) there was a meeting with the health minister and NAAS/GAAS this day, where NAAS presented a preliminary report			
03.06	FDA holds emergency meeting			
03.06	GhanaWeb	http://www.ghanaweb.com/GhanaHomePage/NewsArchive/artikel.php?ID=360701	http://web.archive.org/web/20150815232325/http://www.ghanaweb.com/GhanaHomePage/NewsArchive/artikel.php?ID=360701	FDA holds meeting on Ebola vaccination trials
03.06	Modern Ghana	http://www.modernghana.com/news/621048/1/fda-holds-meeting-on-ebola-vaccination-trials.html	http://web.archive.org/web/20150809184735/http://www.modernghana.com/news/621048/1/fda-holds-meeting-on-ebola-vaccination-trials.html	FDA holds meeting on Ebola vaccination trials
07.06	NDC issues press release where they say no to Ebola trials in the Volta region.			
08.06a	Starr FM Online	http://www.starrfmonline.com/1.4294638	http://web.archive.org/web/20150611033541/http://www.starrfmonline.com:80/1.4294638	NDC says no to “needless” Ebola vaccines trial in Volta region
08.06a	GhanaWeb	http://www.ghanaweb.com/GhanaHomePage/NewsArchive/artikel.php?ID=361484	http://web.archive.org/web/20150816151139/http://www.ghanaweb.com/GhanaHomePage/NewsArchive/artikel.php?ID=361484	Ebola vaccine trial won’t harm Ghanaians – FDA
08.06	Modern Ghana	http://www.modernghana.com/news/621918/1/ndc-says-no-to-needless-ebola-vaccines-trial-in-vr.html	http://web.archive.org/web/20150810104450/http://www.modernghana.com/news/621918/1/ndc-says-no-to-needless-ebola-vaccines-trial-in-vr.html	NDC Says No To Needless Ebola Vaccines Trial In V/R
08.06b	Starr FM Online	http://www.starrfmonline.com/1.4322726	http://web.archive.org/web/20150730025947/http://www.starrfmonline.com/1.4322726	‘Ebola vaccine trial won’t harm Ghanaians’ – FDA
08.06c	Starr FM Online	http://www.starrfmonline.com/1.4313070	http://web.archive.org/web/20150730025658/http://www.starrfmonline.com/1.4313070	Ebola vaccines trial: Scientists confused over ‘Starter’ and ‘Booster’ drugs
08.06b	GhanaWeb	http://www.ghanaweb.com/GhanaHomePage/NewsArchive/artikel.php?ID=361405	http://web.archive.org/web/20150816153837/http://www.ghanaweb.com/GhanaHomePage/NewsArchive/artikel.php?ID=361405	NDC says no to “needless” Ebola vaccines trial in V/R
08.06	An FDA News release is issued, containing an approval of the trial. Mentioned in GhanaWeb on the 10th.			
09.06	Vibeghana (TV)	http://vibeghana.com/2015/06/09/no-ghanaian-should-be-a-guinea-pig-for-ebola-experiments/	http://web.archive.org/web/20160126224127/http://vibeghana.com/2015/06/09/no-ghanaian-should-be-a-guinea-pig-for-ebola-experiments/	No Ghanaian should be a guinea pig for Ebola experiments
09.06a	Modern Ghana	http://www.modernghana.com/news/622229/1/students-paid-for-ebola-drugs-trial.html	http://web.archive.org/web/20150810143334/http://www.modernghana.com/news/622229/1/students-paid-for-ebola-drugs-trial.html	Students Paid For Ebola Drugs Trial
09.06b	Modern Ghana	http://www.modernghana.com/news/622266/1/no-ghanaian-should-be-a-guinea-pig-for-ebol.html	http://web.archive.org/web/20150810143552/http://www.modernghana.com/news/622266/1/no-ghanaian-should-be-a-guinea-pig-for-ebol.html	No Ghanaian should be a guinea pig for Ebola experiments
09.06	GhanaWeb	http://www.ghanaweb.com/GhanaHomePage/NewsArchive/artikel.php?ID=361643	http://web.archive.org/web/20150816181512/http://www.ghanaweb.com/GhanaHomePage/NewsArchive/artikel.php?ID=361643	GSK, Johnson & Johnson behind Ebola vaccine trial in Ghana
09.06	Minister of Health, Alex Segbefia, has directed that the Ebola vaccination trial to be held in the Volta Region be suspended.			
10.06	Parliament discusses the Ebola trials. It is unclear whether the trials are suspended by the house, or if this really is Segbefia’s decision			
10.06a	GhanaWeb	http://www.ghanaweb.com/GhanaHomePage/NewsArchive/artikel.php?ID=361848	http://web.archive.org/web/20150817054520/http://www.ghanaweb.com/GhanaHomePage/NewsArchive/artikel.php?ID=361848	Ebola vaccine trial: FDA exposes Health Ministry
10.06a	Modern Ghana	http://www.modernghana.com/news/622549/1/govt-suspends-ebola-vaccine-trial.html	http://web.archive.org/web/20150810194735/http://www.modernghana.com/news/622549/1/govt-suspends-ebola-vaccine-trial.html	Govt Suspends Ebola Vaccine Trial
10.06b	Modern Ghana	http://www.modernghana.com/news/622538/1/ebola-vaccine-trial-in-vr-suspended.html	http://web.archive.org/web/20150810194914/http://www.modernghana.com/news/622538/1/ebola-vaccine-trial-in-vr-suspended.html	Ebola Vaccine trial in VR suspended
10.06c	Modern Ghana	http://www.modernghana.com/news/622582/1/fda-gives-approval-for-ebola-vaccine-clinical-tria.html	http://web.archive.org/web/20150810182219/http://www.modernghana.com/news/622582/1/fda-gives-approval-for-ebola-vaccine-clinical-tria.html	FDA gives approval for Ebola Vaccine Clinical Trials
10.06b	GhanaWeb	http://www.ghanaweb.com/GhanaHomePage/NewsArchive/artikel.php?ID=361853	http://web.archive.org/web/20150817034559/http://www.ghanaweb.com/GhanaHomePage/NewsArchive/artikel.php?ID=361853	Parliament orders for suspension of Ebola Vaccine trial
10.06c	GhanaWeb	http://www.ghanaweb.com/GhanaHomePage/NewsArchive/artikel.php?ID=361806	http://web.archive.org/web/20150817062832/http://www.ghanaweb.com/GhanaHomePage/NewsArchive/artikel.php?ID=361806	Health Minister suspends Ebola vaccine trial
10.06d	GhanaWeb	http://www.ghanaweb.com/GhanaHomePage/NewsArchive/artikel.php?ID=361789	http://web.archive.org/web/20150817052606/http://www.ghanaweb.com/GhanaHomePage/NewsArchive/artikel.php?ID=361789	FDA okays Ebola for phone vaccine trial
10.06a	Starr FM Online	http://www.starrfmonline.com/1.4385506	http://web.archive.org/web/20150730085150/http://www.starrfmonline.com/1.4385506	Parliament summons health minister over Ebola Vaccine trial
10.06e	GhanaWeb	http://www.ghanaweb.com/GhanaHomePage/NewsArchive/artikel.php?ID=361831	http://web.archive.org/web/20150817033332/http://www.ghanaweb.com/GhanaHomePage/NewsArchive/artikel.php?ID=361831	Mahama’s incompetence makes me regret criticizing Mills’
10.06d	Modern Ghana	http://www.modernghana.com/news/622642/1/im-ready-for-ebola-vaccine-trial-ndc-mp.html	http://web.archive.org/web/20150810174618/http://www.modernghana.com/news/622642/1/im-ready-for-ebola-vaccine-trial-ndc-mp.html	I’m ready for Ebola vaccine trial - NDC MP
10.06b	Starr FM Online	http://www.starrfmonline.com/1.4384597	http://web.archive.org/web/20150730101102/http://www.starrfmonline.com/1.4384597	Relief at Hohoe Midwifery College as gov’t suspends Ebola vaccine trial
10.06c	Starr FM Online	http://www.starrfmonline.com/1.4359741	http://web.archive.org/web/20150730094225/http://starrfmonline.com/1.4359741	Gov’t suspends Ebola vaccine trial
10.06	Modern Ghana	http://www.modernghana.com/news/622425/1/fda-okays-ebola-for-phone-vaccine-trial.html	http://web.archive.org/web/20150810194834/http://www.modernghana.com/news/622425/1/fda-okays-ebola-for-phone-vaccine-trial.html	FDA Okays Ebola For Phone Vaccine Trial
11.06a	GhanaWeb	http://www.ghanaweb.com/GhanaHomePage/NewsArchive/artikel.php?ID=361978	http://web.archive.org/web/20150817082252/http://www.ghanaweb.com/GhanaHomePage/NewsArchive/artikel.php?ID=361978	Mahama should take Ebola vaccine trials to Bole – NPP Chair
11.06b	GhanaWeb	http://www.ghanaweb.com/GhanaHomePage/NewsArchive/artikel.php?ID=362043	http://web.archive.org/web/20150817084202/http://www.ghanaweb.com/GhanaHomePage/NewsArchive/artikel.php?ID=362043	No need to entertain Ebola vacine trial – Deputy Speaker
11.06	Starr FM Online	http://www.starrfmonline.com/1.4391140	http://web.archive.org/web/20150730122430/http://www.starrfmonline.com/1.4391140	Take “cruel” Ebola trial to Bole; Be fair to V/R - NPP reg chair to Mahama
11.06a	Modern Ghana	http://www.modernghana.com/news/622700/1/mps-angry-over-ebola-for-phone-trial.html	http://web.archive.org/web/20150810213743/http://www.modernghana.com/news/622700/1/mps-angry-over-ebola-for-phone-trial.html	MPs Angry Over Ebola For Phone Trial
11.06b	Modern Ghana	http://www.modernghana.com/news/622811/1/ebola-vaccine-trial-could-revolutionize-ghanas-eco.html	http://web.archive.org/web/20150810221820/http://www.modernghana.com/news/622811/1/ebola-vaccine-trial-could-revolutionize-ghanas-eco.html	Ebola vaccine trial could revolutionize Ghana’s economy
11.06c	Modern Ghana	http://www.modernghana.com/news/622801/1/no-to-ebola-vaccine.html	http://web.archive.org/web/20150810213901/http://www.modernghana.com/news/622801/1/no-to-ebola-vaccine.html	No To Ebola Vaccine
11.06c	GhanaWeb	http://www.ghanaweb.com/GhanaHomePage/NewsArchive/artikel.php?ID=362036	http://web.archive.org/web/20150817072107/http://www.ghanaweb.com/GhanaHomePage/NewsArchive/artikel.php?ID=362036	Rejection of Ebola Vaccine trial a sad day for science – Prof. Dodoo
11.06	Vibeghana (TV)	http://vibeghana.com/2015/06/11/parliament-calls-for-suspension-stoppage-of-ebola-vaccine-trial/	http://web.archive.org/web/20160127034105/http://vibeghana.com/2015/06/11/parliament-calls-for-suspension-stoppage-of-ebola-vaccine-trial/	Parliament calls for suspension, stoppage of Ebola vaccine trial
11.06d	GhanaWeb	http://www.ghanaweb.com/GhanaHomePage/NewsArchive/artikel.php?ID=361893	http://web.archive.org/web/20150817074605/http://www.ghanaweb.com/GhanaHomePage/NewsArchive/artikel.php?ID=361893	Test Ebola vaccine on gov’t officials – Lawyer
11.06d	Modern Ghana	http://www.modernghana.com/news/622775/1/rejection-of-ebola-vaccine-trial-a-sad-day-for-sci.html	http://web.archive.org/web/20150810221743/http://www.modernghana.com/news/622775/1/rejection-of-ebola-vaccine-trial-a-sad-day-for-sci.html	Rejection of Ebola Vaccine trial a sad day for science - Prof. Dodoo
11.06e	GhanaWeb	http://www.ghanaweb.com/GhanaHomePage/NewsArchive/artikel.php?ID=362064	http://web.archive.org/web/20150817085500/http://www.ghanaweb.com/GhanaHomePage/NewsArchive/artikel.php?ID=362064	Shut up! – Prof Dodoo tells MPs, others
11.06f	GhanaWeb	http://www.ghanaweb.com/GhanaHomePage/NewsArchive/artikel.php?ID=361872	http://web.archive.org/web/20150817083518/http://www.ghanaweb.com/GhanaHomePage/NewsArchive/artikel.php?ID=361872	I’m ready for Ebola vaccine trial - NDC MP
11.06	Ghanaian Times	http://www.ghanaiantimes.com.gh/house-orders-secret-ebola-vaccination-stopped/	http://web.archive.org/web/20170727181709/http://www.ghanaiantimes.com.gh/house-orders-secret-ebola-vaccination-stopped/	House orders ‘secret’ Ebola vaccination stopped - The Ghanaian Times
11.06 g	GhanaWeb	http://www.ghanaweb.com/GhanaHomePage/NewsArchive/artikel.php?ID=361895	http://web.archive.org/web/20150613192335/http://www.ghanaweb.com/GhanaHomePage/NewsArchive/artikel.php?ID=361895	Gov’t donating Ghanaians to the slaughter house – Atik
11.06 h	GhanaWeb	http://www.ghanaweb.com/GhanaHomePage/features/artikel.php?ID=361775	http://web.archive.org/web/20150817065704/http://www.ghanaweb.com/GhanaHomePage/features/artikel.php?ID=361775	No Ghanaian should be a guinea pig for Ebola experiments
12.06	NAAS/GAAS posts press statement (http://citifmonline.com/2015/06/12/govt-was-warned-against-ebola-vaccine-trial-academy-of-arts-and-sciences/#sthash.2j4TiUiG.dpbs. Archived at: https://web.archive.org/web/20150614005137/http://citifmonline.com/2015/06/12/govt-was-warned-against-ebola-vaccine-trial-academy-of-arts-and-sciences/)			
12.06a	Modern Ghana	http://www.modernghana.com/news/622919/1/where-are-the-cholera-vaccines.html	http://web.archive.org/web/20150811000029/http://www.modernghana.com/news/622919/1/where-are-the-cholera-vaccines.html	Where Are The Cholera Vaccines?
12.06a	GhanaWeb	http://www.ghanaweb.com/GhanaHomePage/NewsArchive/artikel.php?ID=362084	http://web.archive.org/web/20150817114033/http://www.ghanaweb.com/GhanaHomePage/NewsArchive/artikel.php?ID=362084	Noguchi damns Parliament over Ebola vaccine trial
12.06b	GhanaWeb	http://www.ghanaweb.com/GhanaHomePage/entertainment/artikel.php?ID=362193	http://web.archive.org/web/20150817100255/http://www.ghanaweb.com/GhanaHomePage/entertainment/artikel.php?ID=362193	Ebola Vaccine trial: Afia Schwarzenegger blasts FDA
12.06b	Modern Ghana	http://www.modernghana.com/news/622929/1/ghanaians-to-beware-of-the-gove.html	http://web.archive.org/web/20150811012317/http://www.modernghana.com/news/622929/1/ghanaians-to-beware-of-the-gove.html	Ghanaians To Beware Of The Governmental Ebola Vaccine Trials
13.06a	GhanaWeb	http://www.ghanaweb.com/GhanaHomePage/features/artikel.php?ID=362264	http://web.archive.org/web/20150919105802/http://www.ghanaweb.com/GhanaHomePage/features/artikel.php?ID=362264	Ghanaians must be happy to be part of ebola vaccine trial.
13.06	Modern Ghana	http://www.modernghana.com/news/623134/1/may-be-ghanaians-are-ready-for.html	http://web.archive.org/web/20150811035520/http://www.modernghana.com/news/623134/1/may-be-ghanaians-are-ready-for.html	May Be, Ghanaians Are Ready For Mass Immunization Of An Ebola Vaccine That Was Never Tried On Humans
13.06b	GhanaWeb	http://www.ghanaweb.com/GhanaHomePage/features/artikel.php?ID=362263	http://web.archive.org/web/20150817130218/http://www.ghanaweb.com/GhanaHomePage/features/artikel.php?ID=362263	Ghanaians to Beware of the Governmental “Ebola Vaccine Trials”
15.06a	GhanaWeb	http://www.ghanaweb.com/GhanaHomePage/features/artikel.php?ID=362631	http://web.archive.org/web/20150818000541/http://www.ghanaweb.com/GhanaHomePage/features/artikel.php?ID=362631	Monkeying around with an Ebola vaccine
15.06b	GhanaWeb	http://www.ghanaweb.com/GhanaHomePage/NewsArchive/artikel.php?ID=362622	http://web.archive.org/web/20150818014800/http://www.ghanaweb.com/GhanaHomePage/NewsArchive/artikel.php?ID=362622	Health Minister hot over Ebola Vaccine trial deal
15.06c	GhanaWeb	http://www.ghanaweb.com/GhanaHomePage/NewsArchive/artikel.php?ID=362512	http://web.archive.org/web/20150818015006/http://www.ghanaweb.com/GhanaHomePage/NewsArchive/artikel.php?ID=362512	Noguchi boss criticises suspension of Ebola vaccine trials
15.06	Modern Ghana	http://www.modernghana.com/news/623582/1/we-are-not-guinea-pigs-.html	http://web.archive.org/web/20150811105954/http://www.modernghana.com/news/623582/1/we-are-not-guinea-pigs-.html	We Are Not Guinea Pigs!
16.06	Parliament discusses the Ebola trials			
16.06a	Modern Ghana	http://www.modernghana.com/news/623885/1/ebola-vaccine-trial-in-ghana-will-not-spread-virus.html	http://web.archive.org/web/20150811132640/http://www.modernghana.com/news/623885/1/ebola-vaccine-trial-in-ghana-will-not-spread-virus.html	Ebola vaccine trial in Ghana will not spread virus- Health Minister
16.06a	Starr FM Online	http://www.starrfmonline.com/1.4539072	http://web.archive.org/web/20150731051150/http://www.starrfmonline.com/1.4539072	Ebola vaccine trial: MoH to start sensitisation exercise Thursday
16.06a	GhanaWeb	http://www.ghanaweb.com/GhanaHomePage/NewsArchive/artikel.php?ID=362828	http://web.archive.org/web/20150818045313/http://www.ghanaweb.com/GhanaHomePage/NewsArchive/artikel.php?ID=362828	Ebola vaccine trial sensitisation begins on Thursday
16.06b	Starr FM Online	http://www.starrfmonline.com/1.4532392	http://web.archive.org/web/20150731052609/http://www.starrfmonline.com/1.4532392	Parliament summons Alex Dodoo over Ebola attack
16.06b	GhanaWeb	http://www.ghanaweb.com/GhanaHomePage/NewsArchive/artikel.php?ID=362780	http://web.archive.org/web/20150818062611/http://www.ghanaweb.com/GhanaHomePage/NewsArchive/artikel.php?ID=362780	Health Minister faces Parliament on botched Ebola vaccines trial
16.06c	GhanaWeb	http://www.ghanaweb.com/GhanaHomePage/NewsArchive/artikel.php?ID=362844	http://web.archive.org/web/20150818065429/http://www.ghanaweb.com/GhanaHomePage/NewsArchive/artikel.php?ID=362844	Parliament summons Alex Dodoo over ‘Ebola attack’
16.06b	Modern Ghana	http://www.modernghana.com/news/623899/1/parliament-summons-alex-dodoo-over-ebola-attack.html	http://web.archive.org/web/20150811133920/http://www.modernghana.com/news/623899/1/parliament-summons-alex-dodoo-over-ebola-attack.html	Parliament Summons Alex Dodoo Over Ebola Attack
16.06c	Starr FM Online	http://www.starrfmonline.com/1.4511176	http://web.archive.org/web/20150731061132/http://www.starrfmonline.com/1.4511176	Health Minister briefs Parliament on botched Ebola vaccines trial
16.06c	Modern Ghana	http://www.modernghana.com/news/623880/1/ebola-vaccine-trial-sensitisation-to-begin-thursda.html	http://web.archive.org/web/20150811133323/http://www.modernghana.com/news/623880/1/ebola-vaccine-trial-sensitisation-to-begin-thursda.html	Ebola vaccine trial sensitisation to begin Thursday
16.06	Ghanaian Times	http://www.ghanaiantimes.com.gh/kudos-to-the-ghana-academy-of-arts-and-sciences/	http://web.archive.org/web/20170727165350/http://www.ghanaiantimes.com.gh/kudos-to-the-ghana-academy-of-arts-and-sciences/	Kudos to the Ghana Academy of Arts and Sciences - The Ghanaian Times
17.06	Ghanaian Times	http://www.ghanaiantimes.com.gh/prof-dodoo-to-be-dragged-before-house/	http://web.archive.org/web/20170727164558/http://www.ghanaiantimes.com.gh/prof-dodoo-to-be-dragged-before-house/	Prof. Dodoo to be dragged before House - The Ghanaian Times
17.06a	Modern Ghana	http://www.modernghana.com/news/624082/1/minister-raps-mps-over-ebola-for-phone.html	http://web.archive.org/web/20150811204225/http://www.modernghana.com/news/624082/1/minister-raps-mps-over-ebola-for-phone.html	Minister Raps MPs Over Ebola For Phone
17.06b	Modern Ghana	http://www.modernghana.com/news/623974/1/professor-dodoo-happy-to-face-parliament-over-igno.html	http://web.archive.org/web/20150811194328/http://www.modernghana.com/news/623974/1/professor-dodoo-happy-to-face-parliament-over-igno.html	Professor Dodoo happy to face parliament over ‘ignorant’ Ebola comment
17.06c	Modern Ghana	http://www.modernghana.com/news/623993/1/prof-dodoo-faces-mps-over-ebola.html	http://web.archive.org/web/20170727171553/https://www.modernghana.com/news/623993/1/prof-dodoo-faces-mps-over-ebola.html	Prof Dodoo Faces MPs Over Ebola
17.06	Vibeghana (TV)	http://vibeghana.com/2015/06/17/ebola-vaccine-test-has-not-started-in-ghana-segbefia/	http://web.archive.org/web/20160127205449/http://vibeghana.com/2015/06/17/ebola-vaccine-test-has-not-started-in-ghana-segbefia/	Ebola vaccine test has not started in Ghana - Segbefia
17.06a	GhanaWeb	http://www.ghanaweb.com/GhanaHomePage/NewsArchive/artikel.php?ID=362957	http://web.archive.org/web/20150818131209/http://www.ghanaweb.com/GhanaHomePage/NewsArchive/artikel.php?ID=362957	Prof. Dodoo happy to face parliament
17.06b	GhanaWeb	http://www.ghanaweb.com/GhanaHomePage/NewsArchive/artikel.php?ID=362956	http://web.archive.org/web/20150818120534/http://www.ghanaweb.com/GhanaHomePage/NewsArchive/artikel.php?ID=362956	Prof. Dodoo summons ‘extremely amusing’ - Noguchi official
17.06d	Modern Ghana	http://www.modernghana.com/news/623980/1/todays-front-pages.html	http://web.archive.org/web/20150811195402/http://www.modernghana.com/news/623980/1/todays-front-pages.html	Today’s Front Pages
17.06c	GhanaWeb	http://www.ghanaweb.com/GhanaHomePage/NewsArchive/Bagbin-tells-Prof-Dodoo-to-test-Ebola-vaccine-first-363005	http://web.archive.org/web/20150818130320/http://www.ghanaweb.com/GhanaHomePage/NewsArchive/Bagbin-tells-Prof-Dodoo-to-test-Ebola-vaccine-first-363005	Bagbin tells Prof Dodoo to test Ebola vaccine first
18.06a	GhanaWeb	http://www.ghanaweb.com/GhanaHomePage/NewsArchive/Let-s-find-permanent-medical-solution-to-Ebola-Segbefia-363156	http://web.archive.org/web/20150818151639/http://www.ghanaweb.com/GhanaHomePage/NewsArchive/Let-s-find-permanent-medical-solution-to-Ebola-Segbefia-363156	Let’s find permanent medical solution to Ebola - Segbefia
18.06b	GhanaWeb	http://www.ghanaweb.com/GhanaHomePage/NewsArchive/Ebola-Vaccine-trial-sensitisation-starts-today-363195	http://web.archive.org/web/20150818171345/http://www.ghanaweb.com/GhanaHomePage/NewsArchive/Ebola-Vaccine-trial-sensitisation-starts-today-363195	Ebola Vaccine trial sensitisation starts today
18.06a	Modern Ghana	http://www.modernghana.com/news/624501/1/there-shall-be-no-wrong-doing-under-our-watch-who-.html	http://web.archive.org/web/20150811225334/http://www.modernghana.com/news/624501/1/there-shall-be-no-wrong-doing-under-our-watch-who-.html	There shall be no wrong doing under our watch: WHO on ebola trial in Ghana
18.06c	GhanaWeb	http://www.ghanaweb.com/GhanaHomePage/NewsArchive/Parliament-summons-Blakk-Rasta-over-wee-comments-363240	http://web.archive.org/web/20150818170904/http://www.ghanaweb.com/GhanaHomePage/NewsArchive/Parliament-summons-Blakk-Rasta-over-wee-comments-363240	Parliament summons Blakk Rasta over wee comments
18.06b	Modern Ghana	http://www.modernghana.com/news/624379/1/let-them-test-the-ebola-vaccine-on-you-first-bagbi.html	http://web.archive.org/web/20150811223021/http://www.modernghana.com/news/624379/1/let-them-test-the-ebola-vaccine-on-you-first-bagbi.html	Let them test the Ebola vaccine on you first - Bagbin tells Prof Dodoo
18.06c	Modern Ghana	http://www.modernghana.com/news/624470/1/statement-to-parliament-on-the-proposed-anti-ebola.html	http://web.archive.org/web/20150811224325/http://www.modernghana.com/news/624470/1/statement-to-parliament-on-the-proposed-anti-ebola.html	Statement to Parliament on the proposed anti-Ebola vaccine clinical trials in Ghana
18.06d	GhanaWeb	http://www.ghanaweb.com/GhanaHomePage/NewsArchive/Ebola-vaccine-test-has-not-started-in-Ghana-Segbefia-363121	http://web.archive.org/web/20150818165935/http://www.ghanaweb.com/GhanaHomePage/NewsArchive/Ebola-vaccine-test-has-not-started-in-Ghana-Segbefia-363121	Ebola vaccine test has not started in Ghana – Segbefia
18.06e	GhanaWeb	http://www.ghanaweb.com/GhanaHomePage/features/Voltarians-Beware-362878	http://web.archive.org/web/20150818181226/http://www.ghanaweb.com/GhanaHomePage/features/Voltarians-Beware-362878	Voltarians Beware!!!
18.06d	Modern Ghana	http://www.modernghana.com/news/624489/1/commentary-the-vaccination-sc.html	http://web.archive.org/web/20170727172320/https://www.modernghana.com/news/624489/1/commentary-the-vaccination-sc.html	Commentary: The Vaccination: Science Or Pseudo-Science?
19.06	Press release from the World Health Organization (WHO) is assuring that the proposed Ebola vaccine trials will not put any Ghanaian at risk of contracting the virus.			
19.06	GhanaWeb	http://www.ghanaweb.com/GhanaHomePage/NewsArchive/Ebola-trial-will-not-harm-Ghanaians-WHO-assures-363426	http://web.archive.org/web/20150819042333/http://www.ghanaweb.com/GhanaHomePage/NewsArchive/Ebola-trial-will-not-harm-Ghanaians-WHO-assures-363426	Ebola trial will not harm Ghanaians – WHO assures
19.06a	Modern Ghana	http://www.modernghana.com/news/624605/1/parliament-summons-blakk-rasta-for-contemptuous-co.html	http://web.archive.org/web/20150812031453/http://www.modernghana.com/news/624605/1/parliament-summons-blakk-rasta-for-contemptuous-co.html	Parliament summons Blakk Rasta for contemptuous comment
19.06b	Modern Ghana	http://www.modernghana.com/news/624675/1/ebola-trial-not-harmful-who-allays-fears.html	http://web.archive.org/web/20150812025129/http://www.modernghana.com/news/624675/1/ebola-trial-not-harmful-who-allays-fears.html	Ebola Trial Not Harmful--WHO Allays Fears
19.06c	Modern Ghana	http://www.modernghana.com/news/624544/1/sacrifice-for-ebola-vaccine-trial-john-jinapor.html	http://web.archive.org/web/20150812025257/http://www.modernghana.com/news/624544/1/sacrifice-for-ebola-vaccine-trial-john-jinapor.html	Sacrifice For Ebola Vaccine Trial - John Jinapor
19.06d	Modern Ghana	http://www.modernghana.com/music/31009/3/blakk-rasta-hot-over-ganja-mps.html	http://web.archive.org/web/20150812031438/http://www.modernghana.com/music/31009/3/blakk-rasta-hot-over-ganja-mps.html	Blakk Rasta Hot Over Ganja MPs
20.06	Vibeghana (TV)	http://vibeghana.com/2015/06/20/ghana-ebola-trials-will-not-be-compromised-clinical-researches/	http://web.archive.org/web/20160128035159/http://vibeghana.com/2015/06/20/ghana-ebola-trials-will-not-be-compromised-clinical-researches/	Ghana: Ebola Trials will not be compromised- Clinical researches
20.06	Modern Ghana	http://www.modernghana.com/news/624896/1/some-mps-were-ignorant-on-ebola-trial-no-2-ways-ab.html	http://web.archive.org/web/20150812073855/http://www.modernghana.com/news/624896/1/some-mps-were-ignorant-on-ebola-trial-no-2-ways-ab.html	Some MPs were ignorant on Ebola trial, no 2 ways about that - Baako
21.06	GhanaWeb	http://www.ghanaweb.com/GhanaHomePage/NewsArchive/Some-MPs-were-ignorant-on-Ebola-trial-Baako-insists-363642	http://web.archive.org/web/20150819132404/http://www.ghanaweb.com/GhanaHomePage/NewsArchive/Some-MPs-were-ignorant-on-Ebola-trial-Baako-insists-363642	Some MPs were ignorant on Ebola trial – Baako insists
22.06a	Ghanaian Chronicle	http://thechronicle.com.gh/letting-my-thoughts-ramble/	http://web.archive.org/web/20170728071234/http://thechronicle.com.gh/letting-my-thoughts-ramble/	Letting My Thoughts Ramble Ghanaian Chronicle
22.06b	Ghanaian Chronicle	http://thechronicle.com.gh/allowing-ebola-vaccine-trial-shows-ndc-insensitivity-chairman-wontumi/	https://web.archive.org/web/20170728074302/http://thechronicle.com.gh/allowing-ebola-vaccine-trial-shows-ndc-insensitivity-chairman-wontumi/	Allowing Ebola Vaccine Trial Shows NDC Insensitivity -Chairman Wontumi Ghanaian Chronicle
22.06	Modern Ghana	http://www.modernghana.com/news/625247/1/ebola-vaccine-trial-lead-scientist-offers-to-be-us.html	http://web.archive.org/web/20150812141310/http://www.modernghana.com/news/625247/1/ebola-vaccine-trial-lead-scientist-offers-to-be-us.html	Ebola vaccine trial: Lead scientist offers to be used for test
23.06a	GhanaWeb	http://www.ghanaweb.com/GhanaHomePage/NewsArchive/Ebola-vaccines-trial-Lead-scientist-offers-to-be-used-for-test-364062	http://web.archive.org/web/20150820003515/http://www.ghanaweb.com/GhanaHomePage/NewsArchive/Ebola-vaccines-trial-Lead-scientist-offers-to-be-used-for-test-364062	Ebola vaccines trial: Lead scientist offers to be used for test
23.06b	GhanaWeb	http://www.ghanaweb.com/GhanaHomePage/NewsArchive/Mahama-reasons-like-a-stone-age-man-Chairman-Wontumi-364059	http://web.archive.org/web/20150819230241/http://www.ghanaweb.com/GhanaHomePage/NewsArchive/Mahama-reasons-like-a-stone-age-man-Chairman-Wontumi-364059	Mahama reasons like a stone age man - Chairman Wontumi
23.06c	GhanaWeb	http://www.ghanaweb.com/GhanaHomePage/NewsArchive/Allowing-Ebola-Vaccine-trial-shows-NDC-insensitivity-Wontumi-364057	http://web.archive.org/web/20150820021855/http://www.ghanaweb.com/GhanaHomePage/NewsArchive/Allowing-Ebola-Vaccine-trial-shows-NDC-insensitivity-Wontumi-364057	Allowing Ebola Vaccine trial shows NDC insensitivity - Wontumi
24.06d	GhanaWeb	http://www.ghanaweb.com/GhanaHomePage/NewsArchive/Parliament-not-averse-to-criticism-Bagbin-364304	http://web.archive.org/web/20151219000216/http://www.ghanaweb.com/GhanaHomePage/NewsArchive/Parliament-not-averse-to-criticism-Bagbin-364304	Parliament not averse to criticism — Bagbin
25.06	Ghanaian Chronicle	http://thechronicle.com.gh/ebola-and-ethics-autopsy-of-failure/	http://web.archive.org/web/20170728072356/http://thechronicle.com.gh/ebola-and-ethics-autopsy-of-failure/	Ebola and ethics: Autopsy of Failure Ghanaian Chronicle
27.06	GhanaWeb	http://www.ghanaweb.com/GhanaHomePage/features/Who-must-answer-to-GAAS-364910	http://web.archive.org/web/20151228014451/http://www.ghanaweb.com/GhanaHomePage/features/Who-must-answer-to-GAAS-364910	Who must answer to GAAS
30.06	Modern Ghana	http://www.modernghana.com/news/626935/1/on-ebola-flap-both-sides-are-wrong.html	http://web.archive.org/web/20150805081948/http://www.modernghana.com/news/626935/1/on-ebola-flap-both-sides-are-wrong.html	On Ebola Flap, Both Sides Are Wrong
30.06	Ghanaian Times	http://www.ghanaiantimes.com.gh/when-scientists-become-too-secretive/	https://web.archive.org/web/20170728073410/http://www.ghanaiantimes.com.gh/when-scientists-become-too-secretive/	When scientists become too ‘secretive’ - The Ghanaian Times
02.07	It becomes clear that Dodoo needs to face parliament after “ignorant”-statement			
02.07	GhanaWeb	http://www.ghanaweb.com/GhanaHomePage/NewsArchive/Prof-Dodoo-faces-Privileges-Committee-of-Parliament-over-Ebola-ignorant-comment-366040	http://web.archive.org/web/20160127160832/http://www.ghanaweb.com/GhanaHomePage/NewsArchive/Prof-Dodoo-faces-Privileges-Committee-of-Parliament-over-Ebola-ignorant-comment-366040	Prof. Dodoo faces Privileges Committee of Parliament over Ebola ignorant comment
02.07	Ghanaian Chronicle	http://thechronicle.com.gh/who-must-answer-to-gaas-the-world-health-organisation-must-respond-to-the-queries-of-the-ghana-academy-of-arts-and-sciences/	http://web.archive.org/web/20170728072617/http://thechronicle.com.gh/who-must-answer-to-gaas-the-world-health-organisation-must-respond-to-the-queries-of-the-ghana-academy-of-arts-and-sciences/	Who Must Answer To GAAS -(The World Health Organisation Must Respond To The Queries Of The Ghana Academy Of Arts and Sciences) Ghanaian Chronicle
02.07	Ghanaian Times	http://www.ghanaiantimes.com.gh/prof-dodoo-to-face-parliament/	https://web.archive.org/web/20170728073911/http://www.ghanaiantimes.com.gh/prof-dodoo-to-face-parliament/	Prof. Dodoo to face Parliament - The Ghanaian Times
02.07	Starr FM Online	http://www.starrfmonline.com/1.4926550	http://web.archive.org/web/20151018033439/http://www.starrfmonline.com/1.4926550	Prof. Dodoo ‘snubs’ Parliament over Ebola attack
03.07	GhanaWeb	http://www.ghanaweb.com/GhanaHomePage/features/On-Ebola-Flap-Both-Sides-Are-Wrong-365443	http://web.archive.org/web/20160128175024/http://www.ghanaweb.com/GhanaHomePage/features/On-Ebola-Flap-Both-Sides-Are-Wrong-365443	On Ebola Flap, Both Sides Are Wrong
03.07	Ghanaian Times	http://www.ghanaiantimes.com.gh/prof-dodoo-fails-to-show-up/	https://web.archive.org/web/20170728074054/http://www.ghanaiantimes.com.gh/prof-dodoo-fails-to-show-up/	Prof. Dodoo fails to show up - The Ghanaian Times
05.07	GhanaWeb	http://www.ghanaweb.com/GhanaHomePage/NewsArchive/Doubts-over-integrity-of-Ebola-trials-366573	http://web.archive.org/web/20160129221421/http://www.ghanaweb.com/GhanaHomePage/NewsArchive/Doubts-over-integrity-of-Ebola-trials-366573	Doubts over integrity of Ebola trials
06.07	Starr FM Online	http://www.starrfmonline.com/1.4994637	http://web.archive.org/web/20151018040525/http://www.starrfmonline.com/1.4994637	Public forum on Ebola vaccine trial opens in Ho today
07.07	Starr FM Online	http://www.starrfmonline.com/1.5012492	http://web.archive.org/web/20151018041412/http://www.starrfmonline.com/1.5012492	Mothers flee from child immunisation over Ebola vaccine trial fears
08.07	Starr FM Online	http://www.starrfmonline.com/1.5051024	http://web.archive.org/web/20151018042225/http://www.starrfmonline.com/1.5051024	Ebola vaccine trials volunteers promised Int’l insurance cover - GHS
08.07	GhanaWeb	http://www.ghanaweb.com/GhanaHomePage/features/Ebola-Vaccine-Components-Do-Not-Cause-Ebola-Disease-367172	http://web.archive.org/web/20150810134504/http://www.ghanaweb.com/GhanaHomePage/features/Ebola-Vaccine-Components-Do-Not-Cause-Ebola-Disease-367172	Ebola Vaccine Components Do Not Cause Ebola Disease!
14.07	Modern Ghana	http://www.modernghana.com/news/629627/1/i-didnt-insult-you-but-if-you-feel-insulted-im-sor.html	http://web.archive.org/web/20150811185341/http://www.modernghana.com/news/629627/1/i-didnt-insult-you-but-if-you-feel-insulted-im-sor.html	I didn’t insult you but if you feel insulted I’m sorry; Prof. Dodoo tells MPs
14.07	Starr FM Online	http://www.starrfmonline.com/1.5213802	http://web.archive.org/web/20151018051109/http://starrfmonline.com/1.5213802	Prof. Dodoo apologises to Parliament
15.07	GhanaWeb	http://www.ghanaweb.com/GhanaHomePage/world/Trials-of-Ebola-vaccines-begin-in-Europe-and-Senegal-368838	http://web.archive.org/web/20150819010237/http://www.ghanaweb.com/GhanaHomePage/world/Trials-of-Ebola-vaccines-begin-in-Europe-and-Senegal-368838	Trials of Ebola vaccines begin in Europe and Senegal
15.07	GhanaWeb	http://www.ghanaweb.com/GhanaHomePage/NewsArchive/MPs-cannot-be-criticised-for-comments-in-the-House-368705	http://web.archive.org/web/20150818182624/http://www.ghanaweb.com/GhanaHomePage/NewsArchive/MPs-cannot-be-criticised-for-comments-in-the-House-368705	‘MPs cannot be criticised for comments in the House’
16.07	Modern Ghana	http://www.modernghana.com/news/630092/1/live-updateround-two-privileges-committee-hears-ca.html	http://web.archive.org/web/20150812165301/http://www.modernghana.com/news/630092/1/live-updateround-two-privileges-committee-hears-ca.html	Live Update: Round Two: Privileges Committee hears case against Prof Dodoo
16.07a	GhanaWeb	http://www.ghanaweb.com/GhanaHomePage/NewsArchive/Prof-Dodoo-eats-back-his-words-369158	http://web.archive.org/web/20150819231335/http://www.ghanaweb.com/GhanaHomePage/NewsArchive/Prof-Dodoo-eats-back-his-words-369158	Prof. Dodoo eats back his words
16.07b	GhanaWeb	http://www.ghanaweb.com/GhanaHomePage/NewsArchive/You-are-completely-ignorant-Lawyer-dares-MPs-369161	http://web.archive.org/web/20150819221331/http://www.ghanaweb.com/GhanaHomePage/NewsArchive/You-are-completely-ignorant-Lawyer-dares-MPs-369161	You are completely ignorant - Lawyer dares MPs
16.07	Starr FM Online	http://www.starrfmonline.com/1.5277489	http://web.archive.org/web/20151018052658/http://www.starrfmonline.com/1.5277489	I don’t object to Ebola vaccine trial’ - Hohoe Chief
17.07a	GhanaWeb	http://www.ghanaweb.com/GhanaHomePage/NewsArchive/Speaker-vetoes-MPS-charge-at-Prof-Dodoo-369214	http://web.archive.org/web/20150820063142/http://www.ghanaweb.com/GhanaHomePage/NewsArchive/Speaker-vetoes-MPS-charge-at-Prof-Dodoo-369214	Speaker vetoes MPS ‘charge’ at Prof Dodoo
17.07b	GhanaWeb	http://www.ghanaweb.com/GhanaHomePage/NewsArchive/Hohoe-welcomes-Ebola-vaccine-trial-369169	http://web.archive.org/web/20150820045037/http://www.ghanaweb.com/GhanaHomePage/NewsArchive/Hohoe-welcomes-Ebola-vaccine-trial-369169	Hohoe welcomes Ebola vaccine trial
17.07	Modern Ghana	http://www.modernghana.com/news/630238/1/hohoe-paramount-chief-welcomes-ebola-vaccine-trial.html	https://web.archive.org/web/20170728073700/https://www.modernghana.com/news/630238/1/hohoe-paramount-chief-welcomes-ebola-vaccine-trial.html	Hohoe Paramount Chief welcomes Ebola vaccine trial in his town
18.07a	GhanaWeb	http://www.ghanaweb.com/GhanaHomePage/features/We-can-t-fight-EBOLA-with-Substance-Abuse-the-Ignorance-is-too-much-369444	http://web.archive.org/web/20150820094544/http://www.ghanaweb.com/GhanaHomePage/features/We-can-t-fight-EBOLA-with-Substance-Abuse-the-Ignorance-is-too-much-369444	We can’t fight EBOLA with Substance Abuse, the Ignorance is too much
18.07b	GhanaWeb	http://www.ghanaweb.com/GhanaHomePage/NewsArchive/We-were-not-told-to-halt-Ebola-Vaccine-trial-FDA-369485	http://web.archive.org/web/20150926200441/http://www.ghanaweb.com/GhanaHomePage/NewsArchive/We-were-not-told-to-halt-Ebola-Vaccine-trial-FDA-369485	We were not told to halt Ebola Vaccine trial – FDA
18.07c	GhanaWeb	http://www.ghanaweb.com/GhanaHomePage/NewsArchive/FDA-gives-evidence-in-Parliament-369401	http://web.archive.org/web/20150820084113/http://www.ghanaweb.com/GhanaHomePage/NewsArchive/FDA-gives-evidence-in-Parliament-369401	FDA gives evidence in Parliament
19.07	GhanaWeb	http://www.ghanaweb.com/GhanaHomePage/features/Of-politics-politicians-and-arrogance-as-MPs-belittle-our-intelligence-369559	http://web.archive.org/web/20150820141131/http://www.ghanaweb.com/GhanaHomePage/features/Of-politics-politicians-and-arrogance-as-MPs-belittle-our-intelligence-369559	Of politics, politicians and arrogance…as MPs belittle our intelligence
22.07	GhanaWeb	http://www.ghanaweb.com/GhanaHomePage/NewsArchive/Parliament-invites-Prof-Dodoo-again-370229	http://web.archive.org/web/20150821115625/http://www.ghanaweb.com/GhanaHomePage/NewsArchive/Parliament-invites-Prof-Dodoo-again-370229	Parliament invites Prof Dodoo again
22.07	Starr FM Online	http://www.starrfmonline.com/1.5391166	http://web.archive.org/web/20151018061259/http://www.starrfmonline.com/1.5391166	Parliament summons apologetic Prof. Dodoo again
22.07a	Modern Ghana	http://www.modernghana.com/news/631141/1/john-jinapor-must-apologize-to-ghanaians-for-insul.html	http://web.archive.org/web/20150927001932/http://www.modernghana.com/news/631141/1/john-jinapor-must-apologize-to-ghanaians-for-insul.html	John Jinapor Must Apologize to Ghanaians for Insulting our Intelligence for His Ebola vaccine Trials
22.07b	Modern Ghana	http://www.modernghana.com/news/631326/1/parliament-summons-apologetic-prof-dodoo-again.html	http://web.archive.org/web/20150814220725/http://www.modernghana.com/news/631326/1/parliament-summons-apologetic-prof-dodoo-again.html	Parliament Summons Apologetic Prof. Dodoo again
22.07	GhanaWeb	http://www.ghanaweb.com/GhanaHomePage/NewsArchive/Parliament-to-determine-Prof-Dodoo-s-fate-Thursday-370087	http://web.archive.org/web/20150821122358/http://www.ghanaweb.com/GhanaHomePage/NewsArchive/Parliament-to-determine-Prof-Dodoo-s-fate-Thursday-370087	Parliament to determine Prof Dodoo’s fate Thursday
23.07	Modern Ghana	http://www.modernghana.com/news/631507/1/judgement-day-prof-dodoo-blakk-rasta-meet-mps-toda.html	http://web.archive.org/web/20150815055738/http://www.modernghana.com/news/631507/1/judgement-day-prof-dodoo-blakk-rasta-meet-mps-toda.html	Judgement Day: Prof Dodoo, Blakk Rasta meet MPs today
23.07a	GhanaWeb	http://www.ghanaweb.com/GhanaHomePage/NewsArchive/Judgement-Day-Prof-Dodoo-Blakk-Rasta-meet-MPs-today-370425	http://web.archive.org/web/20150821172033/http://www.ghanaweb.com/GhanaHomePage/NewsArchive/Judgement-Day-Prof-Dodoo-Blakk-Rasta-meet-MPs-today-370425	Judgement Day: Prof Dodoo, Blakk Rasta meet MPs today
24.07b	GhanaWeb	http://www.ghanaweb.com/GhanaHomePage/NewsArchive/Prof-Dodoo-pardoned-discharged-370790	http://web.archive.org/web/20150821234551/http://www.ghanaweb.com/GhanaHomePage/NewsArchive/Prof-Dodoo-pardoned-discharged-370790	Prof Dodoo pardoned, discharged
24.07	Starr FM Online	http://www.starrfmonline.com/1.5483817	http://web.archive.org/web/20151018062756/http://starrfmonline.com/1.5483817	Parliament pardons Prof. Dodoo
25.07a	Modern Ghana	http://www.modernghana.com/news/632044/1/is-parliament-becoming-a-monster-ghana-connect-ask.html	http://web.archive.org/web/20150815205706/http://www.modernghana.com/news/632044/1/is-parliament-becoming-a-monster-ghana-connect-ask.html	Is Parliament becoming a Monster? Ghana Connect asks
25.07b	Modern Ghana	http://www.modernghana.com/news/631987/1/give-me-275-slaps-8211-blakk-rasta-tells-mps.html	http://web.archive.org/web/20150815213650/http://www.modernghana.com/news/631987/1/give-me-275-slaps-8211-blakk-rasta-tells-mps.html	Give Me 275 Slaps – Blakk Rasta Tells MPs
25.07c	Modern Ghana	http://www.modernghana.com/news/631940/1/professor-dodoo-escapes-wrath-of-parliament.html	https://web.archive.org/web/20170728073814/https://www.modernghana.com/news/631940/1/professor-dodoo-escapes-wrath-of-parliament.html	Professor Dodoo Escapes Wrath Of Parliament
25.07	GhanaWeb	http://www.ghanaweb.com/GhanaHomePage/NewsArchive/Give-me-275-slaps-Blakk-Rasta-tells-MPs-370894	http://web.archive.org/web/20150822030254/http://www.ghanaweb.com/GhanaHomePage/NewsArchive/Give-me-275-slaps-Blakk-Rasta-tells-MPs-370894	Give me 275 slaps – Blakk Rasta tells MPs

## Additional file

Additional file 1: CGIN Press Release - May 29th 2015. (PDF 283 kb)

## Abbreviations

CGIN: Coalition for Ghana’s Independence Now
GAAS: Ghana Academy of Arts and Sciences
Ghana FDA: Ghana Food and Drug Authority
GPEI: Global Polio Eradication Initiative
GSK: GlaxoSmithKline
IRR: Inter-Rater Reliability
Janssen: Janssen Pharmaceutical Companies of Johnson & Johnson
MP: Member of Parliament
NDC: National Democratic Congress
WHO: World Health Organisation

## Footnotes

1
https://www.google.com/alerts

2 PHP (PHP: Hypertext Preprocessor) is an open source general-purpose scripting language that is especially suited for web development. https://www.php.net

3 The document in question was written by Tawiah Evans, a former journalism student who operates the CGIN with a number of other activists based in Accra.

4 “Prof Dodoo, on the airwaves, had described MPs as ignorant for making misleading and unfounded comments on the Ebola vaccine trial in the country following MPs’ opposition to the vaccine trial.” (Modern Ghana,17.06c)

5 200GH₵ is the equivalent of approximately 48USD/44EUR. According to the World Bank, the minimum monthly wage for a 19-year old or apprentice in Ghana was around 28USD (118GH₵) in 2013 (https://web.archive.org/web/20170728085136/http://datatopics.worldbank.org/jobs/country/ghana)
