# Supplementary material for: Controversial Ebola vaccine trials in Ghana: a thematic analysis of critiques and rebuttals in digital news
Source: BMC Public Health. 2017 Aug 7;17:642. doi: 10.1186/s12889-017-4618-8 (PMC5547580; doi:10.1186/s12889-017-4618-8)
Supplement: Additional file 1: — CGIN Press Release - May 29th 2015. (PDF 283 kb) [file 12889_2017_4618_MOESM1_ESM.pdf]

GhanaIndependence
GhanaIndependence

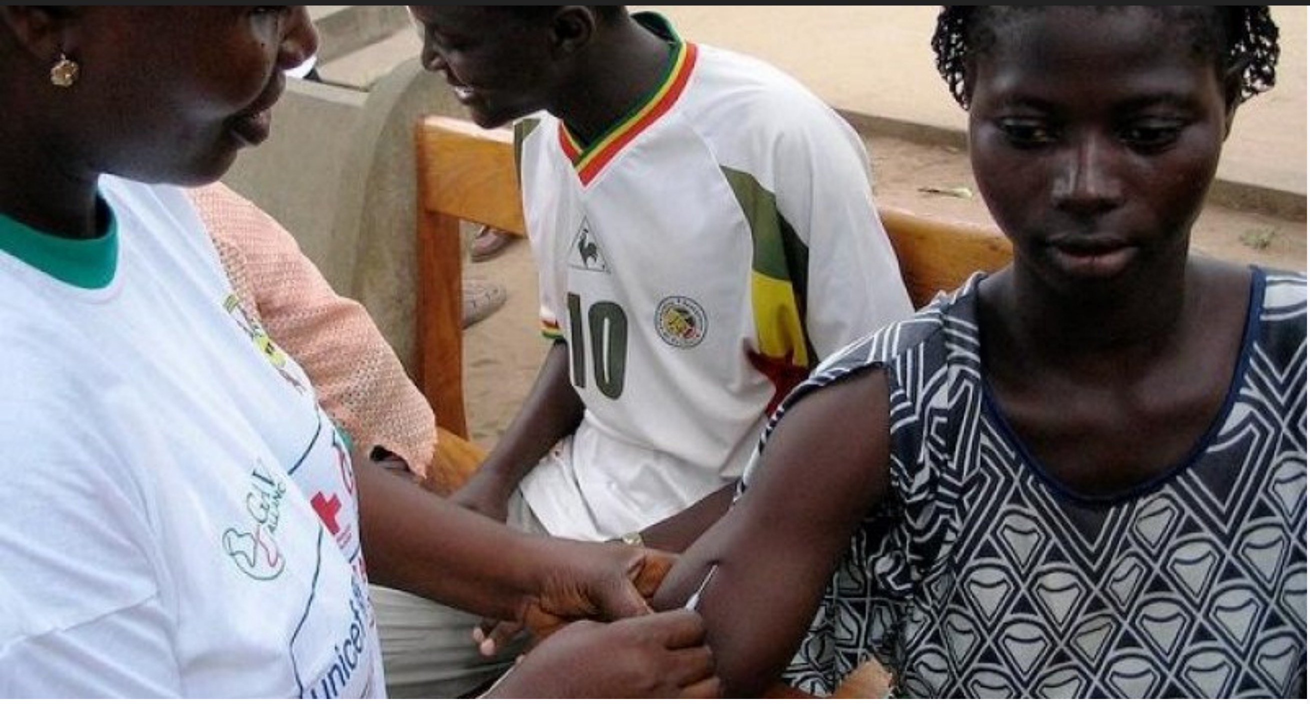

Recent Posts

$700 Million Raw Cash Found In The Home Of Petroleum Minister, Diezani Alison-Madueke

Vatican sacks gay priest after highly public coming out

God does not exist – Wanlov

Weekly Wrap-Up from the Morning Man by Kojo Yankson

Committee probing judicial bribery suspends sitting until November

Press Release

THE GOVERNMENT OF GHANA MUST STOP THE EBOLA VACCINE TESTING IN THE COUNTRY

August 11, 2015 djfuad 0 Comment

The Coalition for Ghana's Independence Now, is disappointed in Ghana as a country for trying to clandestinely selling-out and give up its citizens to be used as Guinea pigs for ebola vaccine trials starting from the Volta region of the country precisely the Hohoe midwifery Training school.

Investigations by Star FM under-cover team have made public a clandestine attempt by authorities to use midwifery students at

Hohoe in the Volta region for human experiment on the ebola vaccine in a country with no ebola case.

We want to say without fear or favor that, ebola is not just a disease but rather a well planed business. A business where people have created an artificial problem and now looking for a market to sell the solution and we are telling Ghanaians beforehand that there is and will be no way by which Ghana can go through this ebola virus human experiment without ebola been spread country wide.

We also would want Ghanaians to know that, since there is no a single case of ebola or patient in Ghana, healthy people would have to be infected with the ebola virus before the said vaccine is administered to them for a gamble.

It is therefore from the above illogical scenario that we consider the intended human experimentation of ebola in Ghana as criminal, human right abuse, thievery and a total disrespect of Ghanaians as human beings.

C.G.I.N is not calling wolf, wolf when there is none but, we are guided by history, logic and critical thinking and our responsibility as citizens to play our role of national security.

Ebola outbreak, which is 100% sure to happen in Ghana should this human trials be allowed to go on, will be the greatest national security threat our country will ever face. The manufactures of the so-called ebola vaccine will look on till a larger number of Ghanaians are killed by the disease here in Ghana before the vaccine will be release and this would happen to make government buy the vaccine at any price.

The American government now owns a patent on new version of the ebola virus called ebobun with patent No. CA2741523A1 and Jonathan S. Towner as the inventor of the latest ebola virus owned by the US government.

At this point, we will want ask our lawyers who have specialized in patent law to tell us the benefits of a patent right over anything. We believe and in-fact convinced by history that, once America have their own version of ebola, they are not going to use any other type of ebola virus to under-take the human experiment

other than ebobun.

Herodotus a man considered to be the father of history once said, “ those who ignore history always condemn themselves for not knowing what is happening at the present moment”.

Historically, the United State of America is notorious for criminal and illegal human experiments and most of these are done with blacks, the most famous one been the Taskegee Syphilis experiment on blacks between the year 1933-1972.

In this un-ethical human experiment, the people involved were not told they were been used for an experiment and where allowed to have sex with their wife just to spread the disease. According to the US public health service, the exercise was meant to last for Six months but was illegally and criminally extended to 40 years.

President Bill Clinton, in 1997 had to render a presidential apology to the victims of the Taskegeeian Syphilis experiment, (**Presidential Apology for the Study at Tuskegeehttp://www.britannica.com**)

“On May 16, 1997, in the East Room of the White House, President Bill Clinton issued a formal apology for the Tuskegee Study of Untreated Syphilis in the Negro Male, the “longest nontherapeutic experiment on human beings” in the history of medicine and public health. That study, conducted under the auspices of the U.S. Public Health Service(PHS) at Tuskegee Institute (now Tuskegee University) in Tuskegee, Alabama, was originally projected to last six months but spanned 40 years—from 1932 to 1972.

The purpose of the study was to determine the effect of untreated syphilis in black men. The men in the study were never told that they had syphilis, a sexually transmitted disease. Instead, government doctors told the men they had “bad blood,” a term that was commonly used to describe a wide range of unspecified maladies”. http://www.britannica.com

Below are former President Bill Clinton’s words of apology to the victims of the criminal experiment “*[They] are a living link to a time not so very long ago that many Americans would prefer not*

to remember but we dare not forget. It was a time when our nation failed to live up to its ideals, when our nation broke the trust…that is the very foundation of our democracy. The United States government did something that was wrong, deeply, profoundly, morally wrong. To the survivors, to the wives and family members, the children and the grandchildren, I say what you know:

No power on Earth can give you back the lives lost, the pain suffered, the years of internal torment and anguish. What was done cannot be undone. But we can end the silence. We can stop turning our heads away. We can look at you in the eye and finally say on behalf of the American people, what the United States government did was shameful, and I am sorry".

As an anti-colonial organization, we see the reactionary apology as a lip service and that is it never America's plan to stop killing people of black origin and Africans in general because, a former secretary of state, Hennery Kissinger in 1974 said, "Depopulation should be the highest foreign policy towards the third worlds.

Bill Gate recently have also declared the next 10 years as a decade of Vaccine and that, he has made available 10 billion US Dollars to create more vaccine to reduce the population in Africa. These and many history and facts call for a serious concern as a group and all individuals to kick against the devilish plans by whoever in charge of the trials.

We further express our disagreement and zero tolerance this planed ebola vaccine test in Hohoe. We see the plans by the ministry of health and the W.H.O as criminal, inhuman, thievery and above all insulting and national human right abuse .

There is no logic in testing a vaccine on a people who do not have the disease in question.

And this is a typical example of why Ghana is not independent and why we should fight our in dependence all anew, and the ministry of health should be ashamed for following the W.H.O,

Tawiah Evans

………………

The convener: Tawiah Evans, The Coalition for Ghana's Independence

0247785986

Evans.tawiah1@gmail.com

← Hello world!

Government must totally stop the ebola vaccine trials and sack all heads of various institutions involved including the minister for defense. →

You May Also Like

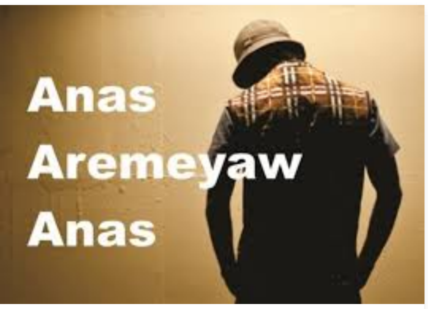

Stopping Anas's videos In Court is Crime against Democracy in the name of Democracy-C.G.I.N

September 15, 2015

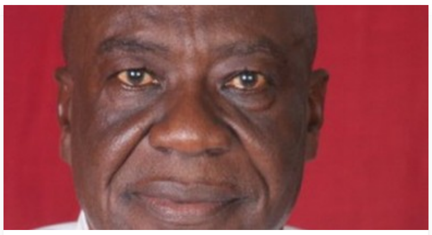

MP for Effiduase Asorkore is practically unfit to represent his people and Ghanaians-C.G.I.N

September 16, 2015

0

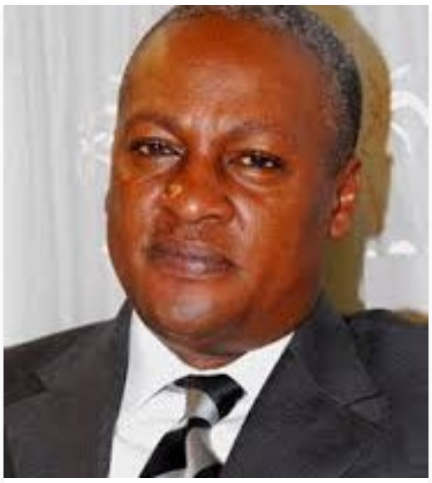

Government must totally stop the ebola vaccine trials and sack all

0

heads of various institutions involved including the minister for defense.

August 11, 2015 0

Leave a Reply

Your email address will not be published. Required fields are marked *

Name *

Email *

Website

Comment

Post Comment
